# HSP27 Provides Stress-Adaptive Proteostasis Buffering in Glioblastoma and Is Therapeutically Vulnerable

**DOI:** 10.3390/cells15191794

**Published:** 2026-09-30

**Authors:** Yuexia Jiao, Qi Liu, Yuchun Zhang, Xiuyuan Wang, Xinjun Liang, Shaozhong Wei, Zijian Tang

**Affiliations:** 1 College of Biomedicine and Health, Huazhong Agricultural University, Wuhan 430070, China; jiao326@webmail.hzau.edu.cn (Y.J.); qiliu@webmail.hzau.edu.cn (Q.L.); zyc123@webmail.hzau.edu.cn (Y.Z.); 2 School of Biomedical Sciences, The Chinese University of Hong Kong, Hong Kong 999077, China; xiuyuanwang@link.cuhk.edu.hk; 3 Hubei Cancer Hospital, Tongji Medical College, Huazhong University of Science and Technology, Wuhan 430074, China; 4 Hubei Provincial Clinical Research Center for Colorectal Cancer, Wuhan 430079, China; 5 Wuhan Clinical Research Center for Colorectal Cancer, Wuhan 430079, China

**Keywords:** HSP27, proteostasis, glioblastoma, translation, stress granule

## Abstract

Glioblastoma, isocitrate dehydrogenase (IDH)-wildtype (GBM), remains one of the most aggressive primary brain tumors, and its proteostasis machinery represents a potential therapeutic target. Here we identify HSP27 as a key regulator-associated proteostasis in glioblastoma, IDH-wildtype, where it is linked to protein synthesis, folding, stress-granule dynamics and ubiquitin-mediated turnover. Ribo-seq showed HSP27 depletion reduced global protein synthesis, while TME labeling coupled to mass spectrometry revealed increased accumulation of unfolded proteins, indicating proteotoxic stress. Proteomic analysis of isolated stress granules (SGs) revealed enhanced sequestration of translational regulators, including EIF4E, mTOR, EIF3M and QKI, into stress granules (SGs), thereby reinforcing translational repression. Ubiquitinome profiling further revealed increased ubiquitination and proteasome-dependent loss of oncogenic and translational regulators, including AKT2, PARP1, EIF4G1, EIF4A3, and DDX5. Functionally, targeting HSP27 inhibits tumor growth and sensitizes glioblastoma to translational blockade. In orthotopic models, antisense oligonucleotide targeting HSP27 in combination with Didemnin B suppressed tumor progression and prolonged survival compared with either single agent. These findings associate HSP27 with proteostasis in glioblastoma, IDH-wildtype, and suggest HSP27-dependent proteostasis buffering as a therapeutic vulnerability.

## 1. Introduction

Glioblastoma, formerly glioblastoma multiforme, is the most aggressive primary brain tumor and accounts for 45–50% of primary malignant brain tumors in adults [1]. Because IDH mutations confer a favorable outcome, the 2021 WHO classification separates glioblastoma, IDH-wildtype (GBM), from IDH-mutant glioblastomas, now classified as astrocytoma, IDH-mutant, WHO grade 4 [2,3]. Despite multimodal treatment combining surgery, radiotherapy, and chemotherapy, median survival for glioblastoma, IDH-wildtype, has remained below two years for decades [4].

One hallmark of glioblastoma, isocitrate dehydrogenase (IDH)-wildtype (GBM) and other malignant cells is their dependence on robust protein homeostasis (proteostasis) to sustain rapid growth, metabolic adaptation, and resistance to stress [5]. Many cancers cells frequently upregulate global protein synthesis to support biomass accumulation and oncogenic signaling [6]. However, enhanced translation load also raises the burden of protein misfolding, aggregation, and degradation stress [6,7]. To survive the pressure, tumor cells coordinate the proteostasis program that includes molecular chaperones, stress granules (SGs), autophagy–lysosomal pathways and the ubiquitin–proteasome system. Emerging evidence suggests that disruption of proteostasis can selectively impair tumor growth and sensitize cancers to therapy [5,7,8,9]. Nevertheless, the mechanisms by which glioblastoma cells integrate translational activity with protein quality control remain incompletely understood.

Heat shock protein 27 (HSP27), encoded by the *HSPB1* gene, is a member of the small heat shock protein family and functions as an ATP-independent molecular chaperone [10,11]. HSP27 participates in diverse cellular processes, including protein folding, cytoskeletal remodeling, oxidative-stress defense, apoptosis resistance, and signal transduction [12,13]. Aberrant HSP27 expression has been reported in multiple malignancies and is commonly associated with aggressive behavior, metastasis, and poor prognosis [14,15]. HSP27 possesses a flexible, intrinsically disordered N-terminal domain (NTD) that is critical for its dynamic oligomerization and ATP-independent chaperone activity, thereby enabling cells to withstand extreme temperatures and maintain proteostasis [16,17,18]. Consequently, HSP27 can participate in the cellular heat-shock response, providing a protective mechanism for maintaining protein homeostasis under extreme temperature conditions. However, whether HSP27 fulfills a similar proteostatic role in glioblastoma, and through which molecular mechanisms, remains largely unexplored.

In the context of glioblastoma, the role of HSP27 remains poorly characterized. Studies have shown that elevated HSP27 expression is associated with malignant transformation and an increased risk of developing several human malignancies, including breast, prostate, gastric, and ovarian cancers, and such overexpression is frequently correlated with poor prognosis [15,19,20,21,22]. Consequently, HSP27 has been proposed as a prognostic marker for various tumor types [14]. Functionally, HSP27 is recognized as a pro-tumorigenic protein that enhances cell survival, promotes metastatic spread, and contributes to drug resistance. For instance, HSP27 can drive epithelial–mesenchymal transition (EMT) and facilitate the metastasis of prostate cancer cells to distant organs [23]. Moreover, as a protein that enhances cell survival, elevated HSP27 expression may be related to cancer drug resistance and the survival capacity during invasion and metastasis. For example, some studies have shown that HSP27 may prevent drugs from binding to microtubules in cancer cells, thereby reducing the sensitivity of cancer cells to therapeutic agents. Pavan and colleagues demonstrated that HSP27 protects microtubules from paclitaxel-induced bundling, and suppression of HSP27 sensitizes ovarian cancer xenografts to paclitaxel treatment [24]. Furthermore, HSP27 has been shown to undergo phosphorylation in response to various microtubule-interfering agents, including vincristine and paclitaxel, suggesting its broad involvement in cellular responses to antimicrotubule chemotherapy [25]. Beyond its role in chemoresistance, HSP27 also promotes cancer cell invasion and metastasis via upstream signaling cascades. For example, upstream molecules such as MAPKAPK2 and MAP2K4 regulate HSP27 expression or phosphorylation, which in turn enhances matrix metalloproteinase-2 (MMP-2) activity and drives prostate cancer cell invasion [26,27]. Collectively, high HSP27 expression in breast, prostate, gastric, and ovarian cancers correlates with increased drug resistance, heightened metastatic potential, and poor patient outcomes. Accordingly, targeting HSP27 has been shown to effectively reduce the tumor burden in these malignancies [28]. Therefore, the regulatory role of HSP27 in tumor cells has become a research hotspot in oncology in recent years. However, the impact of HSP27 on the occurrence, progression, and prognosis of glioblastoma remains unclear at present.

This study extends our understanding of HSP27 functions in glioblastoma from multiple novel perspectives. First, HSP27 depletion broadly suppresses protein translation and profoundly remodels stress granule (SG) composition, highlighting its role for HSP27 in translational control. Second, HSP27 stabilizes multiple oncogenic proteins by limiting the ubiquitin–proteasome system (UPS)-mediated degradation, thereby establishing a direct link between translational regulation and the protein degradation network. Third, combining the antisense oligonucleotide targeting HSP27 (OGX-427) in combination with the protein synthesis inhibitor Didemnin B suppresses glioblastoma growth. Collectively, by employing a multi-omics approach—including Ribo-Seq, protein folding proteomics, SG proteomics, and ubiquitomics—we systematically elucidate the multifaceted roles of HSP27 in maintaining proteostasis in glioblastoma and evaluate the antitumor efficacy of this combined targeting strategy.

## 2. Materials and Methods

### 2.1. Cell Culture, Transfection and Treatment

Human HEK293T, U87-MG and U251-MG cell lines were kindly provided by Prof. Zhiqiang Dong (Huazhong Agricultural University, Wuhan, China). All cell lines were maintained in DMEM (Cytiva, SH30022.01) supplemented with 10% fetal bovine serum (FBS) at 37 °C in a humidified incubator with 5% CO_2_. For siRNA and apatorsen (OGX-427, MCE, HY-145722A)-based interference-based knockdown experiments, cells were transiently transfected for 48 h with small interfering RNAs against *HSPB1* or with OGX-427 using the jetPIME transfection reagent (Polyplus, 101000046). Didemnin B was dissolved in the recommended solvent according to the manufacturer’s instructions and stored at −80 °C until use. Working solutions were freshly prepared from the stock solution before cell treatment. The sequences of individual siRNA and OGX-427 are listed in Supplementary Table S1 of the Supplementary Information.

### 2.2. Cell Counting Kit-8 (CCK-8) Assay

Cell proliferation was assessed using the Cell Counting Kit-8 (CCK-8; GlpBio, GK10001). For transient knockdown experiments, U87-MG and U251-MG cells transfected with NC or siHSP27 were seeded into 96-well plates at a density of 3 × 10^3^ cells per well in 100 μL of culture medium. For stable knockdown experiments, cells were seeded similarly at the same density. For the combined OGX-427 and Didemnin B treatment, cells transiently transfected with OGX-427 for 48 h were seeded into 96-well plates at a density of 3 × 10^3^ cells per well. After cell attachment, the medium was removed and Didemnin B diluted in complete medium was added to the wells. At the indicated time points (24, 48 and 72 h after seeding), 10 μL of CCK-8 solution was added to each well and cells were incubated at 37 °C for 2 h. Absorbance at 450 nm was measured using a microplate reader.

### 2.3. DNA Plasmid Constructs

DNA fragments encoding human *HSPB1* were amplified by PCR from HEK293T cDNA using HS High-Fidelity DNA Polymerase (Abclonal, K20714) and used for subcloning. The amplified *HSPB1* fragment was ligated into the pCDH1 vector using a homologous recombination kit (Yeasen, 10923ES20), with a Flag tag inserted at the C terminus of *HSPB1*. shRNA fragments targeting *HSPB1* were synthesized by TSINGKE Biological Technology Co. (Beijing, China) and subcloned into the linearized pLKO.1 vector. Guide RNA (gRNA) sequences targeting *HSPB1* were synthesized by TSINGKE Biological Technology Co. and ligated into linearized LentiCRISPRv2 (Addgene, #52961). The sequences of individual shRNA are listed in Supplementary Table S1 of the Supplementary Information.

### 2.4. Generation of Stable Expression Cell Lines

To generate stable *HSPB1*-knockdown and *HSPB1*-overexpressing cell lines, psPAX2, pMission-VSVG and the target plasmid were co-transfected into HEK293T cells in 10 cm dishes at a ratio of 4:3:1 for lentiviral packaging. Viral supernatants were collected 48 h after transfection and filtered through 0.45 μm filters to remove cell debris. Viral supernatants were then mixed with a complete DMEM at a 1:1 ratio and added to U87-MG or U251-MG cells for 24 h, followed by selection. Monoclonal cell lines were obtained and validated.

### 2.5. Reverse Transcription Quantitative PCR (RT-qPCR)

Total cellular RNA was extracted using TRIzol reagent (BIOLOGY, Wuhan, China, BOLG601). Reverse transcription was performed using the HiScript III All-in-one RT SuperMix kit (Vazyme, R333-01). mRNA abundance was determined by qPCR using the ChamQ SYBR Color qPCR Master Mix (Vazyme, Q421V23.1), with *GAPDH* used as the internal control. The qPCR program was 95 °C for 30 s, followed by 40 cycles of 95 °C for 5 s and 60 °C for 30 s. The sequences of individual primers are listed in Supplementary Table S2 of the Supplementary Information.

### 2.6. Western Blotting

Protein abundance was analyzed by Western blotting. Total protein was extracted using RIPA buffer (25 mM Tris, pH 7.4, 150 mM NaCl, 1% Triton X-100, 5% glycerol and 1 mM EDTA) supplemented with a protease inhibitor cocktail. After lysis at 4 °C for 30 min, the lysates were centrifuged at 12,000× *g* for 10 min at 4 °C, and the insoluble fractions were discarded. Protein concentrations in the supernatants were determined using the BCA Protein Assay Kit (Thermo Fisher, 23225). Protein samples were then boiled in protein loading buffer for 10 min, separated by SDS-PAGE, and transferred onto PVDF membranes (Vazyme, E802-01), followed by incubation with primary antibodies at 4 °C overnight. The individual antibodies are listed in Supplementary Table S3 of the Supplementary Information. Membranes were then washed three times with TBST (Tween-20) for 10 min each and incubated with horseradish peroxidase (HRP)-conjugated secondary antibody. Signals were visualized using an ECL detection kit (Abbkine, BMU101-CN).

### 2.7. Co-Immunoprecipitation (IP)

MG132-treated cells were thawed on ice and lysed in RIPA buffer containing protease inhibitors (25 mM Tris-HCl, pH 7.4, 150 mM NaCl, 1% Triton X-100, 5% glycerol, and 1 mM EDTA) for 30 min on ice with gentle mixing every 10 min. Lysates were centrifuged at 12,000 rpm for 15 min at 4 °C, and the supernatants were collected for protein quantification using a BCA assay. Equal amounts of total protein (300–400 μg) were incubated with the indicated antibodies overnight at 4 °C under gentle rotation. The following day, pre-equilibrated Protein A/G magnetic beads were added and incubated at room temperature for 1 h. After washing five times with the RIPA buffer, the bound proteins were eluted in 1× loading buffer by boiling at 95 °C for 10 min and subsequently separated by SDS–PAGE. The individual antibodies are listed in Supplementary Table S3 of the Supplementary Information.

### 2.8. Cell Migration Assay

The treated cells were counted and seeded into upper chambers of a 24-well Transwell inserts (8.0 µm pore size, BIOFIL, Guangzhou, China) at a density of 2–3 × 10^4^ cells per well. After incubation at 37 °C in a 5% CO_2_ incubator for 24 h, the medium in the inserts was gently aspirated. Inserts were then washed twice with PBS, fixed with 4% paraformaldehyde (PFA) for 15–20 min, washed once with PBS and stained with 0.1% crystal violet for 15–20 min. Images were captured using a Leica DMi8 inverted microscope. Stained cells were counted using ImageJ, and the data were analyzed and plotted using GraphPad Prism 11.

### 2.9. Establishment of an Orthotopic Glioblastoma Xenograft Model in Nude Mice

The constructed U87-MG cell line was trypsinized, and the digestion was terminated by adding two volumes of complete medium. Cells were gently resuspended in pre-chilled serum-free DMEM, filtered through a 70 μm nylon mesh filter and then counted. The required number of cells was transferred to a new EP tube, centrifuged at 1000× *g* for 5 min and resuspended in the appropriate volume of serum-free DMEM. Mice (BALB/c nude mice, female, 4–6 weeks old) were anesthetized with 2.5% tribromoethanol, and 1 × 10^5^ cells per mouse were inoculated into the striatum. The mouse experiments were approved by the Scientific Ethics Committee of Huazhong Agricultural University (ID Number: HZAUMO-2026-0001).

### 2.10. IVIS Spectrum Small-Animal In Vivo Imaging System

One week after tumor inoculation, mice were intraperitoneally injected with luciferin sodium salt (15 mg/mL). Six minutes later, bioluminescence imaging was performed using the IVIS Spectrum system. For drug treatment, mice that had undergone the first imaging session were evenly divided into four groups and received intraperitoneal injections three times per week as follows: the control mice received normal saline; the OGX-427 group received 5 mg/kg OGX-427; the Didemnin B group received 50 μg/kg Didemnin B; and the combination group (COMB) received both drugs at the same individual doses. Imaging was performed once per week during treatment. After each imaging session, the bioluminescence signal in the mouse brain was quantified using Living Image software, and graphs were generated with the same software. For survival analysis, the day of tumor inoculation was designated day 1, and mice were observed daily. All mice were monitored daily for signs of distress. No specific exclusion criteria were predefined, and no animals or data points were excluded from the final analysis except as noted. Due to the nature of the study, the investigator was not blinded to the group allocation during the experiment. However, all outcome assessments (bioluminescence imaging quantification and tumor volume measurements) were based on objective, software-generated numerical data using standardized protocols and fixed parameters, minimizing the risk of subjective bias. Statistical analyses were performed on unadjusted raw data without any selective exclusion. Statistical analyses were performed using GraphPad Prism 11.

### 2.11. Ribosome Profiling Sequencing

U87-MG cells were transiently transfected with siRNA and incubated for 48 h. The medium was then removed, and cells were washed once with ice-cold PBS, trypsinized for 1 min and neutralized with two volumes of complete medium. Cells were collected, and an aliquot from the same sample was separated for RNA-seq analysis. The remaining cells were centrifuged at 1000× *g* for 5 min, washed twice with ice-cold PBS and completely aspirated. Cell pellets were immediately snap-frozen in liquid nitrogen. Ribosome extraction and library construction from snap-frozen cells were entrusted to Gene Denovo Biotechnology Co. (Guangzhou, China). Briefly, samples were digested with RNase I (NEB, Ipswich, MA, USA) and DNase I (NEB) to remove DNA and RNA. Ribosome recovery and extraction of ribosome-protected RNA fragments were then performed using the RNA Clean and Concentrator-25 kit (Zymo Research, R1017). Libraries were prepared using the NEBNext Multiple Small RNA Library Prep Set for Illumina (catalog nos. E7300S and E7300L). PCR products of 140–160 bp were enriched to generate cDNA libraries, which were sequenced on an Illumina NovaSeq X Plus by Gene Denovo Biotechnology Co., with a target data output of 6 Gb per sample.

### 2.12. RNA Sequencing and Ribosome Sequencing

RNA-seq paired with Ribo-seq was performed by Genedenovo Biotechnology Co., Ltd. (Guangzhou, China). Total RNA was extracted using the TRIzol method. mRNA was enriched using mRNA Capture Beads and, after bead purification, fragmented at high temperature. Fragmented mRNA was used as the template, and RNA integrity was assessed using an Agilent 2100 Bioanalyzer. After quality control, libraries were constructed using the Hieff NGS Ultima Dual-mode mRNA Library Prep Kit (Yeasen, 12309ES) and sequenced by Genedenovo Biotechnology Co. on an Illumina NovaSeq X Plus platform, with a target data output of 6 Gb per sample.

### 2.13. Ribo-Seq and RNA-Seq Data Analysis

For ribosome profiling (Ribo-seq) and RNA-seq, raw reads were trimmed to remove adapters and low-quality bases using Trimmomatic (v0.39). Clean reads were aligned to the human reference genome (GRCh38) using STAR (v2.7.9a). For Ribo-seq, reads aligned to rRNA, tRNA, and other non-coding RNAs were filtered out, and the remaining reads representing ribosome-protected fragments were used for downstream analysis. Gene-level read counts were quantified using featureCounts (v2.0.3). Differential expression analysis was performed using DESeq2 (v1.36.0). For Ribo-seq data, genes with a fold change (FC) > 1.5 and false discovery rate (FDR) < 0.05 were considered significantly changed at the translational level. For RNA-seq data, genes with FC > 2.0 and FDR < 0.05 were considered significantly changed at the transcriptional level. Gene set enrichment analysis (GSEA) was performed using the clusterProfiler package (v4.4.4) against the Kyoto Encyclopedia of Genes and Genomes (KEGG) and Gene Ontology (GO) databases. All bioinformatics analyses were performed with three biological replicates per condition.

### 2.14. Flow Cytometry

For flow cytometric cell-cycle analysis, U87-MG and U251-MG glioblastoma cells (transiently transfected for 48–72 h or stably constructed) were trypsinized, and digestion was neutralized with two volumes of complete medium. After centrifugation, cells were washed twice with ice-cold PBS, counted and fixed in 75% ethanol overnight at 4 °C. Fixed cells were centrifuged at 1500 rpm for 5 min at 4 °C, washed twice with ice-cold PBS, resuspended in ice-cold PBS and incubated with RNase A (200 μg/mL) and propidium iodide (PI, 50 μg/mL) for 30 min at 37 °C in the dark. Samples were analyzed on a high-sensitivity analytical flow cytometer (CytoFLEX LX). For TME-labeled cells, trypsinization was followed by neutralization with two volumes of cysteine-free DMEM supplemented with 10% FBS and two washes with ice-cold PBS. Cells were resuspended in PBS and analyzed on the same flow cytometer. Excitation and emission wavelengths for TME fluorescence were set to 405 nm and 450 ± 40 nm, respectively. Flow cytometry data were analyzed using FlowJo v10.

### 2.15. TME Labeling of Unfolded Proteins

TME was synthesized by Norbel Biotechnology Co., Ltd. (Shanghai, China). When cells reached 80% confluence, the culture medium was removed, and the cells were washed twice with PBS. Cells were then incubated with 50 μM TME (diluted from a 1 mM stock solution in DMSO using cysteine-free DMEM) for 30 min at 37 °C in a 5% CO_2_ incubator [29]. After TME removal, the cells were washed twice with PBS, and PBS supplemented with protease inhibitors was added. Cells were scraped off on ice using a cell scraper. Collected cell samples were then sonicated at 35% amplitude with 3 s on and 6 s off for a total of 6 min, with the sonication performed on ice throughout. Sonicated lysates were centrifuged at 12,000 rpm for 10 min at 4 °C, and protein concentrations were determined using the BCA Protein Assay Kit (Thermo Fisher, 23225).

### 2.16. Copper-Catalyzed Click Chemistry Reaction and Magnetic Bead Enrichment

TME-labeled cells and vehicle-control cells (treated with an equal volume of DMSO) were prepared in triplicate as described above. A total of 400 µg of cell lysate was used for lysate analysis. The copper-catalyzed azide–alkyne cycloaddition (CuAAC) reaction was initiated by mixing the following components to the indicated final concentrations: 1 mM CuSO_4_/THPTA (1:5 dilution), 5 mM aminoguanidine hydrochloride, 0.25 mM biotin azide, 2 mg/mL cell lysate, and 5 mM sodium ascorbate [29]. The reaction mixture was then incubated on a rotator for 2 h in the dark. Proteins that had undergone the click reaction were then enriched. Briefly, 40 μL of 100% trichloroacetic acid (TCA) was added to the protein reaction mixture, which was immediately vortexed and incubated on ice for 30 min to precipitate proteins completely. The mixture was centrifuged at 15,000× *g* for 15 min at 4 °C. The supernatant was discarded, and the pellet was washed once with absolute ethanol, followed by centrifugation at 15,000× *g* for 15 min at 4 °C. The protein pellet was dissolved in 60 μL of 8 M urea by pipetting and diluted using a gradient dilution method. The diluted protein solution was centrifuged at 14,000× *g* for 15 min at 4 °C, and the supernatant was transferred to a new microcentrifuge tube. Streptavidin magnetic beads (30 μL; Dynabeads Streptavidin) were added, and the mixture was incubated overnight on a rotator at 4 °C. After incubation, beads were washed three times with wash buffer for 5 min per wash and then washed three times with 50 mM TEAB (triethylammonium bicarbonate). Bead-enriched proteins were sent to Jingjie Biotechnology Co., Ltd. (Hangzhou, China) for separation using an EASY-nLC 1200 ultra-high-performance liquid chromatography system.

### 2.17. Native PAGE Gel Electrophoresis

For extraction of high molecular weight (HMW) aggregated proteins, cells were lysed in HMW lysis buffer (50 mM Tris-HCl, pH 7.4, 150 mM NaCl, 1% NP-40 and 0.1% sodium deoxycholate) supplemented with protease inhibitor cocktail. After lysis on ice for 30 min, the lysates were centrifuged at 16,000× *g* for 15 min at 4 °C. Supernatant were transferred to new tubes, and protein concentration were determined using a BCA kit (Thermo Fisher). Then, 50–100 μg of protein was gently mixed with an equal volume of 2× non-denaturing protein loading buffer. Samples were resolved by 3–8% Tris-Acetate PAGE (Sangon Biotech) at 150 V for 3 h, with the electrophoresis apparatus placed in an ice-water bath throughout the run. After electrophoresis, proteins were transferred onto a membrane using glycine transfer buffer at 110 V for 2.5 h.

### 2.18. Stress Granule Isolation

SGs were isolated as previously described [30]. Briefly, when cells reached 80–90% confluence, culture medium in 15 cm dishes was replaced with fresh complete medium. Cells were then scraped at room temperature. Cells from three 15 cm dishes were collected into a 15 mL centrifuge tube and centrifuged at 1500× *g* for 3 min at room temperature. The supernatant was removed as thoroughly as possible, and the cell pellet was immediately snap-frozen in liquid nitrogen and stored at −80 °C. Cell collection was completed within 10 min. For SG isolation, cells were placed on ice for 5 min and lysed in 800 μL of lysis buffer (50 mM Tris, pH 7.6, 50 mM NaCl, 5 mM MgCl_2_, 0.1% NP-40, 1 mM beta-mercaptoethanol, 1× EDTA-free protease inhibitor cocktail, 1 mM Na_3_VO_4_ and 1× NaF). The lysate was passed 5–6 times through a 25 G needle syringe on ice and incubated on a rotator at 4 °C for 15 min. Protein concentration was determined using the BCA method. An aliquot of lysate was retained as input. Equal amounts of protein from the remaining lysate were transferred to new tubes and centrifuged at 1000× *g* for 5 min at 4 °C to remove nuclei. The supernatant was centrifuged at 18,000× *g* for 20 min at 4 °C to pellet SGs, and the soluble supernatant (cytoplasmic fraction) was transferred to new tubes. The SG pellet was washed twice with lysis buffer by gentle resuspension, resuspended in 600 μL of lysis buffer, passed three times through an insulin syringe and centrifuged at 850× *g* for 2 min at 4 °C. The resulting supernatant was transferred to a new tube.

### 2.19. Survival Analysis Using the CGGA Database

mRNA expression data and corresponding clinical information were obtained from the CGGA database (mRNAseq_325 dataset). Patients with IDH-wildtype, glioblastoma (GBM) were selected for analysis. *HSPB1* expression levels were used to stratify patients into high- and low-expression groups based on the median expression value. Kaplan–Meier survival curves were generated, and the log-rank test was used to compare the overall survival between the two groups. Hazard ratios (HRs) and 95% confidence intervals (CIs) were calculated using Cox proportional hazards regression. All survival analyses and curve plotting were performed using GraphPad Prism 11 (GraphPad Software, San Diego, CA, USA).

### 2.20. Bioinformatics Analysis of Public Datasets

*HSPB1* mRNA expression data and corresponding clinical information were obtained from public databases, including TCGA_GBM, CGGA, Gravendeel, and Rembrandt, via the Gliovis platform (http://gliovis.bioinfo.cnio.es/, accessed on 17 July 2026). For survival analysis, patients with glioblastoma, IDH-wildtype (GBM) were stratified by the *HSPB1* expression level using upper quartile cutoffs as indicated. Kaplan–Meier survival curves were generated, and the log-rank test was used to compare overall survival between groups. Hazard ratios (HRs) and 95% confidence intervals (CIs) were calculated using Cox proportional hazards models.

### 2.21. Protein Expression and Survival Analysis of the TCGA-GBM Cohort

To evaluate the prognostic value of HSPB1 at the protein level in glioblastoma, we retrieved the reverse-phase protein array (RPPA) data of HSPB1 (annotated as HSP27) and corresponding clinical information for the TCGA-GBM cohort from The Cancer Proteome Atlas (TCPA) database (https://tcpaportal.org/tcpa/, accessed on 17 July 2026). Patients were stratified into HSPB1-high- and HSPB1-low-expression groups based on the median protein expression value (n = 88 for both groups). Kaplan–Meier survival curves were generated, and the statistical significance between the two groups was assessed using the log-rank (Mantel–Cox) test. A *p* value < 0.05 was considered statistically significant.

### 2.22. Statistical Analysis

Overall survival (OS) was analyzed using the Kaplan–Meier method, and differences between survival curves were assessed by the log-rank test. Univariate and multivariate Cox proportional hazards regression analyses were performed to identify independent prognostic factors associated with patient outcomes. Unless otherwise specified, all statistical tests were two-sided. A *p* value < 0.05 or an adjusted *p* value (P.adjust) < 0.05 after multiple-testing correction using the Benjamini–Hochberg method was considered statistically significant. Statistical analysis was performed using GraphPad Prism (version 11). Comparisons between two groups were conducted using two-tailed unpaired Student’s *t*-tests. For time-course studies, including cell-growth and tumor-growth curves, comparisons were performed using two-way ANOVA. A *p* value < 0.05 was considered statistically significant. No specific study protocol was pre-registered for this exploratory study, as it did not involve a confirmatory clinical intervention or a predefined hypothesis-testing design requiring prospective registration. All experimental procedures were conducted following standard laboratory protocols and were documented in real-time laboratory notebooks.

## 3. Results

### 3.1. HSP27 Promotes Glioblastoma Proliferation and Migration In Vitro and In Vivo

**Figure 1:**
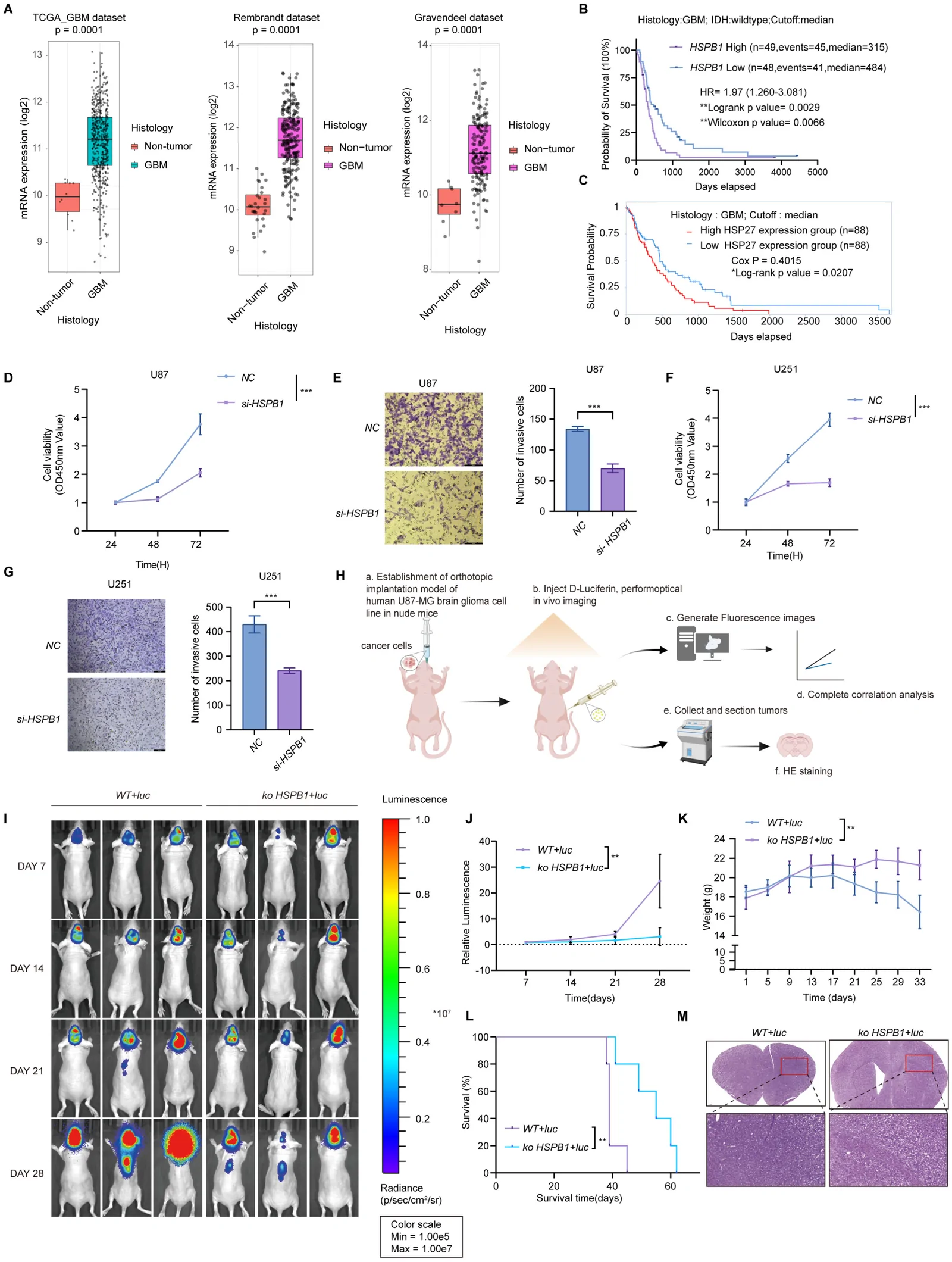
**HSP27 promotes glioblastoma proliferation and migration in vitro and in vivo.** (**A**) HSPB1 mRNA expression levels in GBM versus non-tumor brain tissues across three independent datasets: TCGA_GBM, Rembrandt, and Gravendeel. Data are presented as box plots with individual data points; boxes indicate median and interquartile range. Statistical significance was determined using the Mann–Whitney U test (*p* < 0.0001 for all comparisons). (**B**) Kaplan–Meier overall survival curves for patients with IDH-wildtype GBM from the CGGA database, stratified by HSPB1 expression level (high vs. low, median cutoff). Median survival: 315 vs. 484 days; HR = 1.97, 95% CI 1.260–3.081; log-rank test *p* = 0.0029. (**C**) Kaplan–Meier overall survival curves for GBM patients from TCGA, stratified by HSP27 protein expression level (median cutoff). High-expression group: n = 88; low-expression group: n = 88; log-rank test *p* = 0.0207. Panels (**B**,**C**) represent two independent validation cohorts showing consistent prognostic value of HSPB1 in GBM. (**D**,**F**) CCK-8 assays measuring cell viability in U87-MG and U251-MG cells following siRNA treatment. Cell viability was assessed at the indicated time points (24, 48, and 72 h) after transfection. Statistical significance was determined using two-way ANOVA followed by Šidák’s post hoc test. (**E**,**G**) Transwell migration assays evaluating the effect of HSPB1 knockdown on glioblastoma cell migration. Scale bar, 100 μm. Statistical significance was determined using a two-tailed unpaired Student’s *t*-test. (**H**) Schematic illustration of the establishment of an orthotopic glioblastoma xenograft model by intracranial implantation of luciferase-labeled U87-MG cells into nude mice. The diagram in H was created using BioGDP (https://www.biogdp.com). (**I**) Representative bioluminescence images of mice at 7, 14, 21 and 28 days after tumor implantation. The experiment included 5 mice per group (WT + Luc and ko HSPB1 + Luc), and the images display three mice per group for each time point. (**J**) Quantification of bioluminescence signals showing tumor burden. Statistical significance was determined by two-way repeated measures (RM) ANOVA followed by Tukey’s post hoc test. (**K**) Tumor-growth curves quantified from bioluminescence imaging signal intensities. Data are presented as mean ± SEM (n = 5 mice per group). Statistical significance was determined by two-way RM ANOVA. (**L**) Body weight was measured starting from the day of tumor implantation (day 1) and subsequently every 4 days thereafter. Data are presented as mean ± SEM (n = 5 mice per group). Statistical significance was determined by two-way RM ANOVA. M) Kaplan–Meier survival curves of nude mice bearing U87-MG-Luc (WT + Luc) or HSP27-KO U87-MG-Luc (HSP27-KO + Luc) tumors. Survival was compared using the log-rank test (n = 5 mice per group; *p* = 0.0072). (**M**) Representative H&E-stained for tumors in the nude mice. Data information: Data are presented as mean ± SEM unless otherwise stated. n represents the number of independent biological replicates or mice as indicated. *p* < 0.05 was considered statistically significant. ns means not significant; * means *p* < 0.05. ** means *p* < 0.01. *** means *p* < 0.001. HR, hazard ratio; CI, confidence interval.

To characterize the expression pattern of *HSPB1*/HSP27 in glioblastoma (GBM), we first analyzed multiple independent datasets using the Gliovis platform. As shown in Figure 1A, *HSPB1* mRNA expression was significantly higher in GBM tissues than in non-tumor brain tissues across three datasets: TCGA_GBM, Rembrandt, and Gravendeel (all *p* = 0.0001). Given the elevated expression of *HSPB1* in GBM, we next evaluated its impact on patient survival. In two independent cohorts—the CGGA database and the Gravendeel dataset—high *HSPB1* expression was consistently associated with significantly shorter overall survival in patients with IDH-wildtype GBM. In the CGGA cohort, median survival was 315 days for the high-expression group versus 484 days for the low-expression group (HR = 1.97, 95% CI 1.260–3.081; log-rank *p* = 0.0029; Figure 1B). Consistent with this, in the Gravendeel dataset, high *HSPB1* expression also predicted worse survival, with a median survival of 10.9 months for the high-expression group (n = 45) versus 15.5 months for the low-expression group (n = 136) in patients with IDH-wildtype GBM (HR = 0.69, 95% CI 0.49–0.98 for low versus high expression; log-rank *p* = 0.0356; Supplementary Figure S1A). Together, these findings from two independent cohorts demonstrate that high *HSPB1* expression is robustly associated with poor prognosis in IDH-wildtype GBM. Furthermore, we assessed the prognostic value of HSP27 protein levels using TCGA data. Patients with high HSP27 protein expression (n = 88) had significantly shorter overall survival than those with low expression (n = 88) (log-rank *p* = 0.0207; Figure 1C). These results demonstrate that *HSPB1*/HSP27 is highly expressed in GBM and that its elevated expression is significantly associated with poor patient prognosis, suggesting that *HSPB1*/HSP27 may serve as a prognostic biomarker and a potential therapeutic target in glioblastoma, IDH-wildtype (GBM).

**Figure 2:**
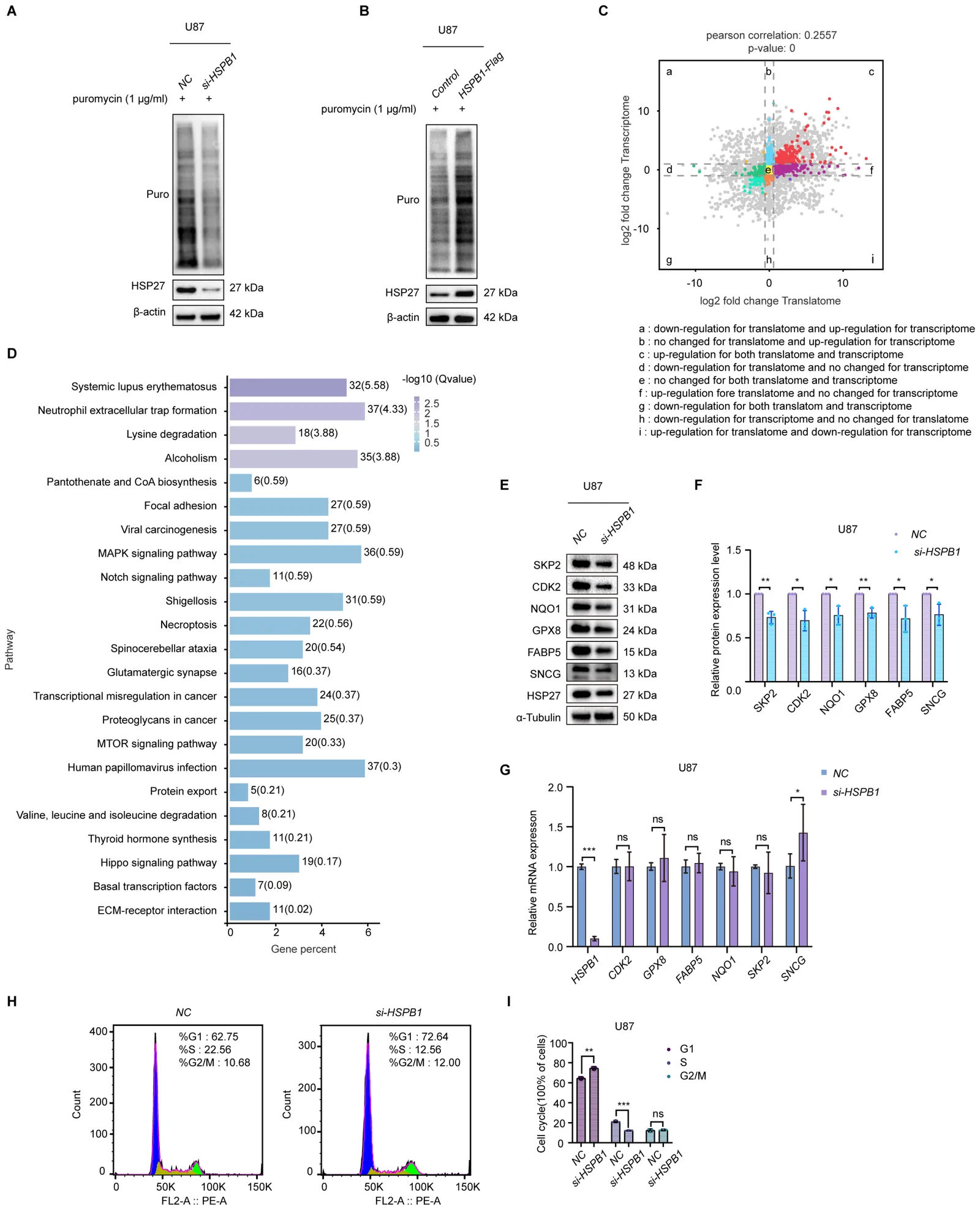
HSP27 globally enhances protein translation in glioblastoma cells. (**A**,**B**) Puromycin incorporation assays in U87-MG cells after HSPB1 knockdown (**A**) or HSP27 overexpression (**B**), detected by anti-puromycin Western blotting. (**C**) Integrated Ribo-seq and RNA-seq analysis after *HSPB1* knockdown (differential thresholds: FC > 1.5 and FDR < 0.05 for Ribo-seq; FC > 2 and FDR < 0.05 for RNA-seq). (**D**) KEGG pathway enrichment analysis of 1661 genes with translatome efficiency independent of transcriptional changes after HSP27 depletion. (**E**) Western blotting validation of selected candidate proteins (CDK2, SKP2, GPX8, NQO1, FABP5, and SNCG) in U87-MG cells. (**F**) Quantification of the relative protein expression level. (**G**) Relative mRNA levels of the same candidates examined by qRT-PCR. (**H**) Flow cytometric analysis of cell-cycle distribution in U87-MG cells. (**I**) Quantification of the percentage of cells in G1, S and G2/M phases from H. Data information: Data are presented as mean ± SEM from three independent biological replicates *n* = 3 for panels (**F**,**G**,**I**). Statistical significance for panels (**F**,**G**,**I**) was determined using a two-tailed unpaired Student’s *t*-test. ns means not significant; * means *p* < 0.05, ** means *p* < 0.01, *** means *p* < 0.001.

In vitro functional assays further revealed that *HSPB1* knockdown significantly suppressed the proliferation and migration of U87-MG and U251-MG glioblastoma cells (Figure 1D–G and Figure S1B–I), whereas HSP27 overexpresses these phenotypes in U87-MG cells (Supplementary Figure S1J–L). To investigate whether HSP27 is required for glioblastoma maintenance in vivo, we established an orthotopic xenograft model using luciferase-expressing U87-MG cells with stable *HSPB1* knockdown or control (Figure 2H and Figure S2A,B). Consistent with our in vitro findings, bioluminescence imaging showed that *HSPB1* knockdown significantly suppressed intracranial tumor growth [31] (Figure 1I,J and Figure S2C). Moreover, tumor-bearing mice in the wild-type (WT) group exhibited a pronounced loss in body weight during the late stage of the experiment (after day 19), whereas this reduction was significantly mitigated in the *HSPB1* knockout group (Figure 1K). More importantly, the Kaplan–Meier survival analysis demonstrated a statistically significant extension of overall survival in the knockout group relative to controls (Figure 1L). Hematoxylin and eosin (H&E) staining confirmed the presence of densely cellular, infiltrative intracranial lesions, thereby validating successful model establishment (Figure 1M). Collectively, these in vivo data establish that HSP27 depletion, while not curative, confers a meaningful survival advantage and delays glioblastoma progression in mice, reinforcing its potential as a therapeutic target.

### 3.2. HSP27 Globally Enhances Protein Translation in Glioblastoma Cells

Previous studies have showed that HSP27 specifically binds to EIF4G, a key scaffolding protein of the translation initiation complex, under heat shock conditions, thereby regulating cellular translation [32]. To determine whether HSP27 controls protein synthesis under non-heat-shock conditions, we performed puromycin incorporation assays. *HSPB1* knockdown reduced global protein synthesis in U87-MG and U251-MG cells (Figure 2A and Figure S3A), whereas HSP27 overexpression increased protein synthesis in U87-MG cells (Figure 2B). To assess whether HSP27 regulates gene expression at the translational level, we integrated ribosome profiling (Ribo-seq) and RNA-seq data from control and HSP27-deficient glioblastoma cells. Translational and transcriptional changes were weakly correlated after HSP27 depletion (Pearson r = 0.2557; Figure 2C). Nine-quadrant analysis further showed that most differentially expressed genes fell in quadrants a, b, d, f, h, and i, which represent the 1661 genes with translational efficiency without matching transcriptional changes; only a limited subset of genes showed concordant translatome and transcriptome changes (quadrants c and g). KEGG enrichment analysis of the 1661 transcription-independent genes identified mTOR signaling, protein export, MAPK signaling, focal adhesion, Hippo signaling, and ECM–receptor interaction pathways (Figure 2D). Thus, HSP27-mediated translational control affects oncogenic signaling, protein homeostasis and tumor-associated cellular functions, and HSP27 loss causes a broad reduction in translation efficiency.

We next sought to identify genes with reduced translation but unchanged transcript abundance after HSPB1 knockdown. Given our previous findings that *HSPB1* knockdown significantly inhibited GBM cell proliferation, we hypothesized that its translationally regulated targets might be enriched in pathways governing cell proliferation and metabolism. Therefore, we prioritized genes functionally linked to these processes. A panel of genes associated with cell-cycle progression and metabolism, including CDK2, SKP2, GPX8, NQO1, FABP5, and SNCG, was selected for validation [33,34,35,36,37]. These genes were prioritized based on their established roles in driving glioblastoma cell proliferation, thereby ensuring that the validated targets are functionally relevant to the malignant phenotype of GBM. Western blotting analysis revealed that the protein levels of these candidates were markedly reduced following HSP27 depletion (Figure 2E,F and Figure S3B,C), whereas their mRNA levels were unchanged (Figure 2G and Figure S3D), consistent with the integrated Ribo-seq and RNA-seq data. To preliminarily validate the downstream effects of the aforementioned translational regulation at the protein level, we analyzed the correlation between HSPB1 and GPX8 in the clinical CPTAC GBM dataset. A significant positive correlation was observed (Spearman r = 0.44, *p* = 5.072 × 10^−6^; Supplementary Figure S3E), further supporting the proposed regulatory mechanism. Given that both CDK2 and SKP2 are involved in the regulation of G1/S transition, with CDK2 acting as a cyclin-dependent kinase that promotes G1/S progression and SKP2 affecting S phase entry by regulating the ubiquitin-mediated degradation of cell cycle inhibitors [38], the present study further investigated the impact of HSP27 depletion on the cell-cycle distribution of glioblastoma cells. Flow cytometric analysis confirmed that *HSPB1* knockdown reduced the proportion of cells in S phase and induced G1 phase arrest (Figure 2H,I and Figure S3F). Collectively, these findings demonstrate that HSP27 promotes global protein synthesis and selectively sustains the translation of key regulators involved in cell-cycle progression (e.g., CDK2 and SKP2), thereby facilitating glioblastoma cell proliferation and G1/S transition.

### 3.3. HSP27 Supports Protein Folding and Prevents Unfolded-Protein Accumulation in Glioblastoma Cells

**Figure 3:**
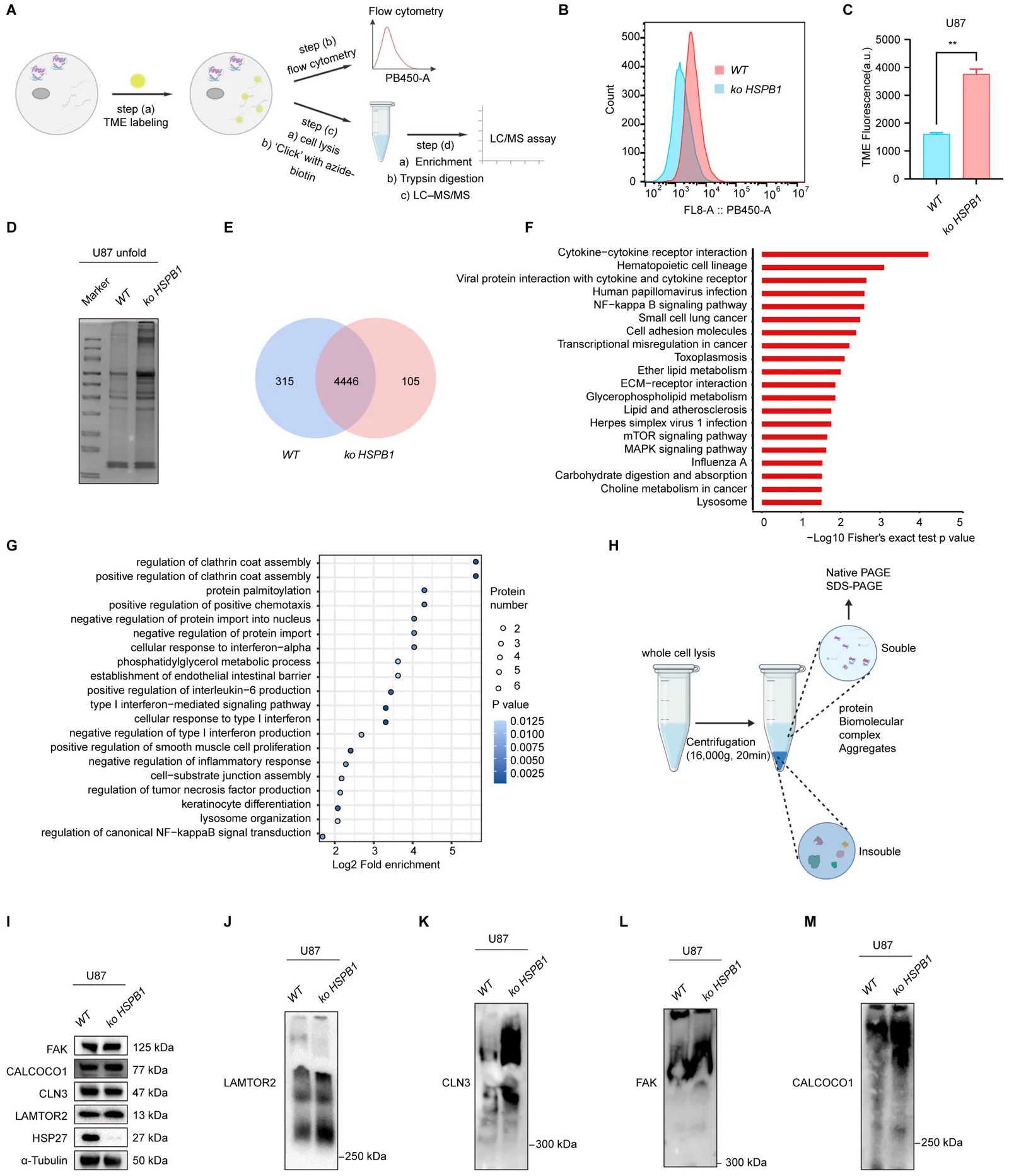
**HSP27 supports protein folding and prevents unfolded-protein accumulation in glioblastoma cells.** (**A**) Schematic of the TME-labeling strategy for detecting unfolded proteins. (**B**) Representative flow cytometry histograms showing TME fluorescence intensity in control and HSP27-depleted cells. Cells were incubated with TME (50 μM) for 30 min. (**C**) Quantification of TME fluorescence intensity from (**B**). Data are presented as mean ± SEM from three independent biological replicates. Statistical significance was determined by unpaired *t* test, ** means *p* < 0.01. (**D**) Silver staining of TME-labeled unfolded proteins enriched by click chemistry, showing increased unfolded-protein abundance after HSP27 depletion. (**E**) Venn diagram of mass spectrometry identified TME-labeled unfolded proteins in control and *HSPB1* knockout (KO) cells, showing 105 proteins specifically enriched in the KO group. (**F**) KEGG pathway enrichment analysis of the 105 specifically enriched unfolded proteins in *HSPB1* KO cells. (**G**) Gene Ontology (GO) biological process analysis of the same 105 proteins. (**H**) Schematic of cell-lysate fractionation based on protein solubility. (**I**) Western blotting analysis of LAMTOR2, CLN3, CALCOCO1 and FAK in soluble fractions separated by SDS-PAGE. (**J**–**M**) Western blotting analysis of the indicated proteins in soluble fractions separated by native PAGE.

A canonical function of HSP27 is its ATP-independent molecular chaperone activity in protein folding and proteostasis maintenance. To examine this activity, we used TME (50 μM), a probe that labels unfolded proteins (Figure 3A). Flow cytometry showed that *HSPB1* knockdown significantly increased TME fluorescence (Figure 3B,C and Figure S4A), indicating accumulation of unfolded proteins. Enrichment of TME-labeled proteins by click chemistry followed by silver staining confirmed increased unfolded-protein abundance after HSP27 depletion (Figure 3D). These data show that HSP27 helps prevent unfolded-protein accumulation and is required to maintain protein-folding homeostasis in glioblastoma cells. To assess whether HSP27 loss triggers compensatory upregulation of other heat shock proteins, we examined HSP105, HSP90, HSP70, HSP60, HSP40, and HSP10 by Western blotting. In both U87 and U251 cells, *HSPB1* knockdown efficiently reduced HSP27 but did not alter the expression of these HSP family members (Supplementary Figure S4B,C). These data indicate that HSP27 depletion does not induce a general heat-shock response or compensatory chaperone upregulation.

To further characterize the impact of *HSPB1* knockdown on proteostasis, we performed proteomic sequencing of enriched TME-labeled unfolded proteins. This analysis identified 105 proteins specifically upregulated after *HSPB1* knockdown (Figure 3E). Functional enrichment of these proteins showed significant enrichment of lysosome, MAPK signaling, mTOR signaling, NF-κB signaling, cytokine–cytokine receptor interaction and ECM–receptor interaction pathways, indicating that HSP27 depletion induces a coordinated stress-adaptive program involving proteostasis regulation, inflammatory signaling and cell-adhesion remodeling (Figure 3F). Gene Ontology analysis similarly identified lysosome organization, regulation of canonical NF-κB signaling, type I interferon-mediated signaling, cell–substrate junction assembly, clathrin coat assembly and lipid metabolic processes (Figure 3G). These findings suggest that HSP27 depletion disrupts protein-folding homeostasis and dysregulates degradation pathways and membrane-trafficking systems. Following the unbiased screening of HSP27-associated protein folding networks, we prioritized candidates specifically involved in the degradation of misfolded proteins. This targeted approach allows us to specifically validate the hypothesis that HSP27 mediates its oncogenic effects by regulating the clearance of misfolded proteins in GBM cells. Immunoblotting was then used to validate the aggregation status of selected proteins among the 105 specifically upregulated unfolded proteins (Figure 3H). Following *HSPB1* knockdown in U87-MG and U251-MG cells, LAMTOR2 (a protein associated with lysosomal and mTOR signaling), CLN3 (a lysosomal membrane protein involved in vesicular transport and lysosomal homeostasis), CALCOCO1 (a selective autophagy receptor mediating recognition and degradation of misfolded proteins), and FAK (a core regulator of focal adhesion dynamics and cell–matrix signaling) all exhibited migration patterns above 250 kDa, but SDS-PAGE results showed that the total expression levels of the aforementioned candidate proteins did not change significantly, suggesting that these proteins undergo unfolding and aggregation [39,40] (Figure 3I–M and Figure S4D–H). Together, these data indicate that HSP27 depletion induces proteotoxic stress and activates an adaptive network involving lysosomal and autophagic remodeling, mTOR-associated signaling, inflammatory responses and focal-adhesion reprogramming.

### 3.4. HSP27 Depletion Remodels SGs Composition and Sequesters Translational Regulators

**Figure 4:**
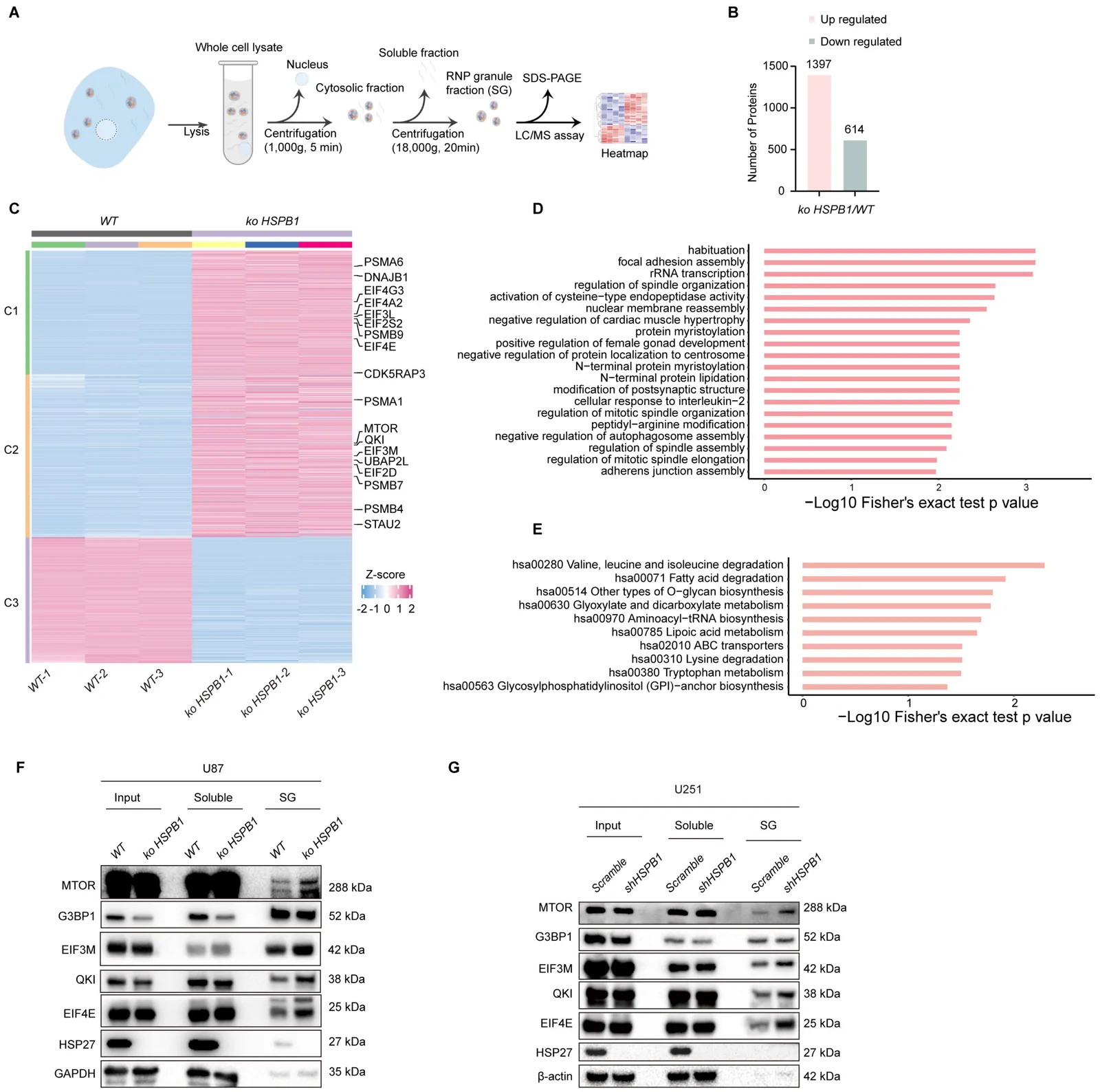
HSP27 depletion remodels SG composition and sequesters translational regulators. (**A**) Workflow for SG isolation and proteomic analysis in control and *HSPB1*-depleted glioblastoma cells. (**B**) Bar plot showing the number of proteins identified and significantly enriched in SGs under control and *HSPB1* knockdown conditions. (**C**) Hierarchical clustering of differentially enriched proteins in SGs after HSP27 depletion. (**D**) Gene Ontology (GO) biological process analysis of proteins significantly upregulated in SGs following *HSPB1* knockdown. (**E**) KEGG pathway enrichment analysis of proteins significantly upregulated in SGs in HSP27-deficient cells. (**F**,**G**) Western blotting analysis of SG fractions showing increased accumulation of EIF4E, mTOR, QKI, and EIF3M upon HSP27 depletion.

SGs are dynamic, reversible assemblies of proteins and RNA that form under stress conditions and support cellular adaptation. Given the proteotoxic stress induced by HSP27 depletion, we asked whether HSP27 influences SG formation and composition. SG-related components were separated and enriched, and the effect of HSP27 deficiency on SG components was analyzed by mass spectrometry (Figure 4A). Mass spectrometry analysis identified significant enrichment of 1397 proteins in SGs following HSP27 depletion, whereas 614 proteins were downregulated in SGs, indicating extensive remodeling of SGs under proteotoxic conditions (Figure 4B). Hierarchical clustering confirmed substantial remodeling of SG-associated proteins following HSP27 depletion (Figure 4C). Notably, multiple translation-related proteins, including EIF4E, EIF4A2, EIF3L, EIF3M, EIF2S2, and EIF2D, were markedly enriched in SGs upon HSP27 loss, suggesting enhanced sequestration of the translational machinery. mTOR and the RNA-binding protein QKI also accumulated in SG fractions from HSP27-deficient cells, indicating disruption of translation-regulatory signaling and RNA metabolism. Bioinformatic analysis demonstrated that the upregulated SG proteins were predominantly associated with biological processes including rRNA transcription, spindle organization, focal adhesion assembly, and regulation of autophagosome assembly, suggesting broad disruption of protein synthesis, mitotic progression, and cellular stress adaptation (Figure 4D). KEGG pathway enrichment further identified significant alterations in aminoacyl-tRNA biosynthesis, ABC transporters, fatty-acid degradation, and branched-chain amino acid metabolism, consistent with metabolic reprogramming during translational stress (Figure 4E). Given the critical role of stress granules (SGs) in sequestering translational machinery and modulating global protein synthesis [41], we selected candidate proteins for validation based on their established functions in translation regulation [42]. We prioritized SG-resident proteins known to interact with translation initiation factors or ribosomal components [43,44,45]. Immunoblotting of isolated SGs confirmed the accumulation of EIF4E, EIF3M, mTOR, and QKI in SGs (Figure 4F,G). Collectively, these results demonstrate that HSP27 deficiency remodels SG composition by aberrantly recruiting translational regulators, RNA-binding proteins, chaperones and proteasome-associated proteins. These changes likely reinforce translational repression and may contribute to proteostasis collapse in glioblastoma cells.

### 3.5. HSP27 Globally Regulates Ubiquitin–Proteasome-Mediated Protein Degradation in Glioblastoma Cells

**Figure 5:**
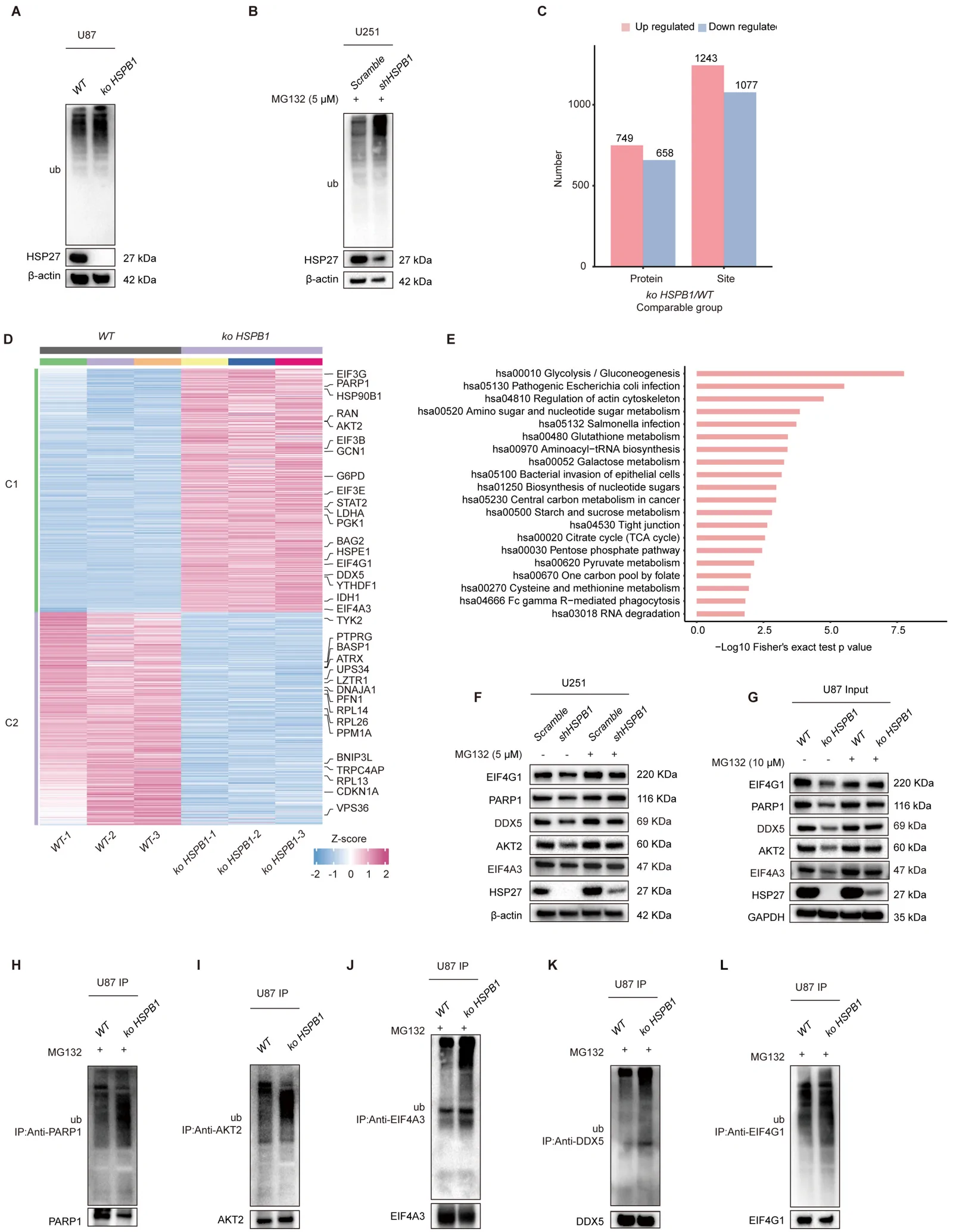
HSP27 globally regulates ubiquitin–proteasome-mediated protein degradation in glioblastoma cells. (**A**,**B**) Immunoblotting analysis showing global ubiquitination levels in Control and HSP27-depleted glioblastoma cells. (**C**) Bar plot summarizing the number of ubiquitinated proteins and ubiquitination sites identified by quantitative ubiquitinome analysis in *Control* and *HSPB1* knockdown groups. (**D**) Hierarchical clustering of differentially ubiquitinated proteins between Control and HSP27-depleted cells. (**E**) KEGG pathway enrichment analysis of proteins with significantly increased ubiquitination after HSP27 depletion. (**F**,**G**) Immunoblotting analysis showing that treatment with the proteasome inhibitor MG132 restores AKT2, EIF4G1, EIF4A3, DDX5, and PARP1 protein levels in HSP27-deficient cells. (**H**–**L**) Immunoprecipitation assays validating the ubiquitination levels of candidate proteins after HSP27 depletion. PARP1 (**H**), AKT2 (**I**), EIF4A3 (**J**), DDX5 (**K**), and EIF4G1 (**L**) were immunoprecipitated, followed by immunoblotting with an anti-ubiquitin antibody.

Given that HSP27 depletion induced translational inhibition, proteotoxic stress, and SG remodeling, we next asked whether HSP27 maintains proteostasis in glioblastoma cells by regulating protein degradation pathways. Immunoblotting showed that *HSPB1* knockdown increased global ubiquitination levels in U87-MG cells (Figure 5A,B). Quantitative ubiquitin proteomics then revealed extensive remodeling of the ubiquitination landscape after HSP27 loss (Figure 5C,D). A large subset of proteins showed increased ubiquitination in HSP27-deficient cells, indicating enhanced ubiquitin-dependent protein turnover (Figure 5D). KEGG enrichment analysis of up-ubiquitinated proteins identified RNA degradation and aminoacyl-tRNA biosynthesis pathways, indicating disrupted RNA homeostasis and translation (Figure 5D). Metabolic and oxidative-stress-related terms were also enriched, suggesting impaired central metabolism and redox buffering. Enrichment of actin cytoskeleton and tight-junction pathways further suggests that HSP27 protects cytoskeletal integrity by limiting ubiquitin-dependent degradation of structural regulators. Collectively, these data demonstrate that HSP27 supports the malignant progression of glioblastoma cells by suppressing the ubiquitination of proteins involved in translation, metabolism, redox homeostasis, and cytoskeletal organization.

Integrating the unbiased ubiquitination profiling data, we filtered candidate proteins based on their functional annotations in DNA damage repair and cell proliferation. This selection strategy ensures that the validated ubiquitination substrates or regulators are mechanistically linked to the established roles of HSP27 in maintaining genomic stability and driving GBM proliferation [46]. Among these candidates, several proteins with established functions in DNA repair and/or cell proliferation were prioritized for validation, including AKT2 [47], PARP1 [48], EIF4A3 [49], EIF4G1 [50], and DDX5 [51]. To determine whether the reduced abundance of these candidate proteins after HSP27 depletion was mediated by proteasomal degradation, we treated glioblastoma cells with the proteasome inhibitor MG132. Immunoblotting showed that HSPB1 knockdown significantly reduced AKT2, PARP1, EIF4A3, EIF4G1, and DDX5 protein levels, whereas MG132 treatment restored their abundance [ref for proteasome restoration approach] (Figure 5F,G), indicating that HSP27 loss promotes proteasome-dependent degradation of these proteins. We then performed co-immunoprecipitation assays to examine the ubiquitination of representative candidates after HSP27 depletion. Consistent with the proteomics data, *HSPB1* knockdown markedly increased the ubiquitination levels of AKT2, PARP1, EIF4A3, EIF4G1, and DDX5 (Figure 5H–L). These findings confirm that HSP27 loss promotes ubiquitination and degradation of multiple proteins involved in oncogenic signaling, DNA repair, RNA metabolism, and translational regulation. Taken together with the observed increase in ubiquitination in the MG132 rescue experiment, these data suggest that HSP27 depletion is associated with enhanced ubiquitination and proteasome-dependent turnover of oncogenic and translational regulators by limiting ubiquitin-mediated proteasomal turnover. Thus, we propose that HSP27 may contribute to the maintenance of key protein networks in glioblastoma cells, at least in part, by restricting ubiquitination and proteasome-mediated degradation of these client proteins.

### 3.6. HSP27 Constitutes a Rational Therapeutic Target for the Treatment of Glioblastoma

**Figure 6:**
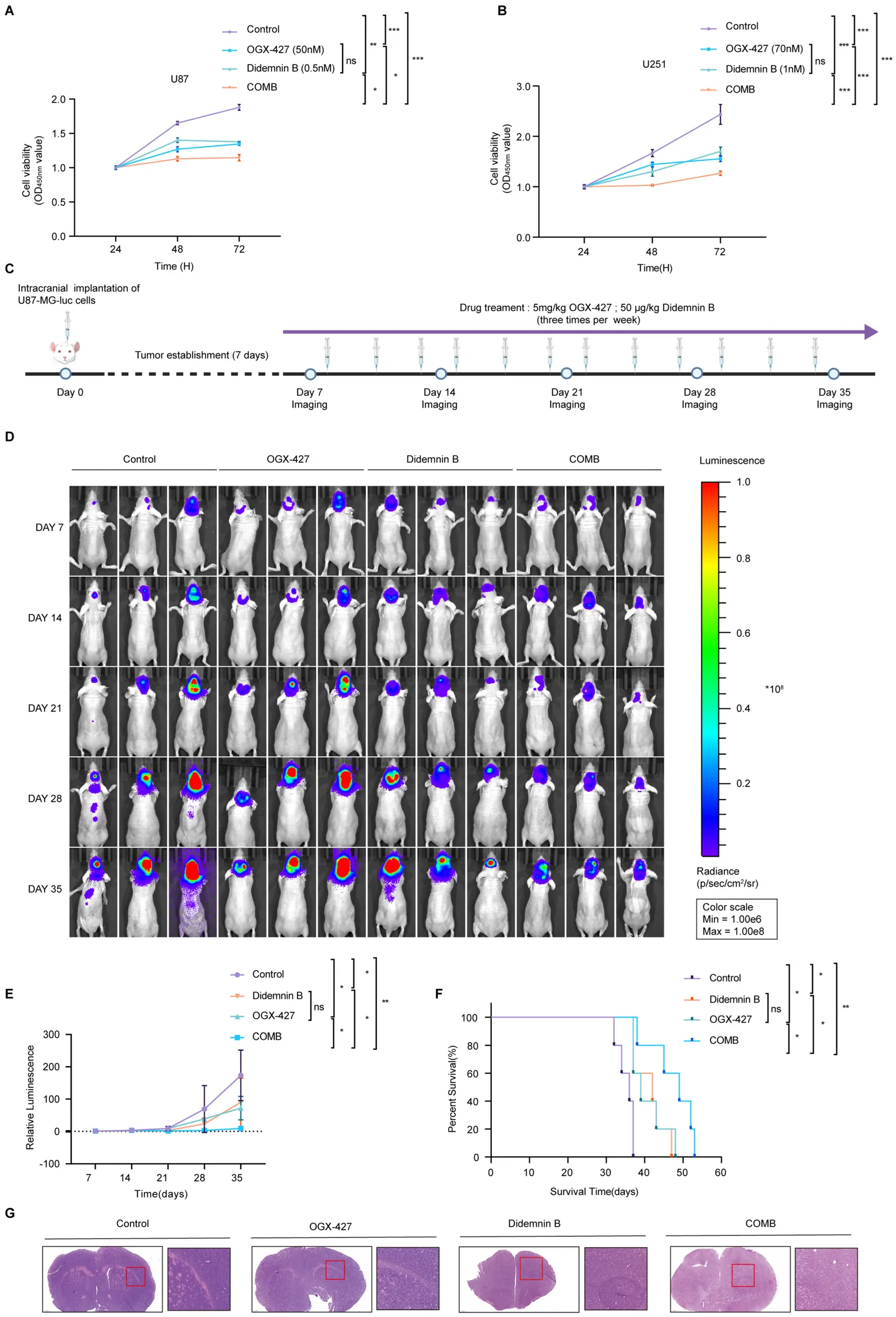
HSP27 constitutes a rational therapeutic target for the treatment of glioblastoma. (**A**,**B**) CCK-8 assays showing the effects of OGX-427 and Didemnin B, alone or in combination, on the proliferation of U87-MG (**A**) and U251-MG (**B**) cells. Statistical significance was determined using two-way ANOVA followed by Tukey’s post hoc test. (**C**) Schematic of the in vivo treatment protocol. (**D**) Representative bioluminescence images of orthotopic glioblastoma-bearing mice under different treatments. The experiment included 5 mice per group (control, OGX-427, Didemnin B, and COMB), and the images display three mice per group for each time point. (**E**) Quantification of bioluminescence signals showing tumor burden. Statistical significance was determined by two-way repeated measures (RM) ANOVA followed by Tukey’s post hoc test. (**F**) Kaplan–Meier survival curves of glioblastoma-bearing mice under different treatment conditions, Survival was compared using the log-rank test (n = 5 mice per group). (**G**) H&E staining of brain tumor sections from each group. Scale bar 500 µm. Data information: Data are presented as mean ± SEM. *n* = 3 biological replicates for panels A and B; *n* = 5 mice per group for panels (**D**–**F**). ns means not significant; * means *p* < 0.05, ** means *p* < 0.01, *** means *p* < 0.001.

Because HSP27 maintains both translational homeostasis and proteostasis, we next tested whether pharmacological HSP27 inhibition could enhance the therapeutic effect of translational blockade. Therefore, in this project, we used an antisense oligonucleotide (ASO) targeting HSP27 mRNA to reduce HSP27 mRNA levels and consequently decrease HSP27 protein expression. ASOs, owing to their relatively small molecular weight, can efficiently cross the blood–brain barrier and have been employed in several clinical trials for glioblastoma [52]. OGX-427 is a 2’-methoxyethyl-modified antisense oligonucleotide that reduces HSP27 expression by targeting its mRNA, and has entered phase II clinical trials for castration-resistant prostate cancer [53]. In addition, we concurrently used the protein translation inhibitor Didemnin B, which suppresses the key translation elongation factor eEF1A1, thereby inhibiting protein synthesis [54]. Of relevance, Didemnin B had previously been evaluated in phase II clinical trials for glioblastoma. Although it was eventually abandoned because of poor therapeutic efficacy, its prior advancement to phase II at least demonstrates that it can cross the blood–brain barrier, an essential feature for any agent targeting glioblastoma [55]. CCK-8 assays performed in U87-MG and U251-MG cells showed that combined treatment with OGX-427 and Didemnin B produced greater growth inhibition than either agent alone, indicating enhanced antiproliferative activity in vitro (Figure 6A,B). To validate the therapeutic potential in vivo, we established an orthotopic glioblastoma model (Figure 6C). Consistent with the in vitro results, OGX-427 monotherapy significantly suppressed intracranial tumor progression compared to the control group (*p* < 0.05), confirming that HSP27 targeting alone exerts a measurable antitumor effect in vivo. More importantly, the combined OGX-427 and Didemnin B dramatically suppressed intracranial tumor progression compared with single-agent treatment (Figure 6D,E). Mice receiving the combination therapy also showed the longest overall survival (Figure 6F). Tumor cells displayed diffuse growth with spindle-like alignment and destruction of adjacent brain parenchyma, accompanied by vacuolar degeneration and perivascular accumulation, confirming successful glioblastoma formation (Figure 6G). Collectively, these results identify HSP27 as a rational therapeutic target and demonstrate that its inhibition potentiates the anti-glioblastoma efficacy of translational blockade.

## 4. Discussion

Glioblastoma cells have high demands for protein synthesis and stress adaptation, which support rapid proliferation in the hostile tumor microenvironment. Proteostasis maintenance is therefore essential for glioblastoma progression [56,57]. In this study, we identify HSP27 as a key regulator associated with proteostasis in glioblastoma, IDH-wildtype, where it is linked to protein translation, protein folding, SG dynamics, and ubiquitin-mediated protein turnover. HSP27 depletion was associated with translational suppression, proteotoxic stress, aberrant SG remodeling, and extensive ubiquitin-dependent degradation of key oncogenic and translational regulators, and with impaired tumor growth in our models. Notably, our study assessed HSP27 protein abundance rather than its phosphorylation or oligomerization state, and the GBM datasets analyses are based on HSPB1 RNA expression; our conclusions are therefore restricted to abundance-dependent HSP27 functions and should be interpreted with caution at the functional level.

HSP27 is classically recognized as an ATP-independent molecular chaperone involved in stress adaptation and protein folding [58]. Our findings extend this understanding by showing that HSP27 is also associated with altered translational homeostasis in glioblastoma, IDH-wildtype. While it is not surprising that HSP27 modulates protein folding given its well-established chaperone activity, our data indicate that HSP27 is also linked to the control of global protein synthesis—a function not previously attributed to this protein. This unexpected observation raises the intriguing possibility that HSP27 couples the processes of translation and folding, thereby integrating two fundamental layers of proteostasis. The precise molecular mechanisms by which HSP27 regulates translation remain unclear and warrant thorough investigation. Two non-mutually exclusive mechanisms could be envisioned. First, HSP27 has been reported to directly associate with translation initiation factors such as EIF4G to modulate protein synthesis. Second, as a molecular chaperone, HSP27 may indirectly support translation by maintaining the folding and function of translation-related proteins; its depletion would thus impair the translational machinery as a consequence of proteotoxic stress. Our current data do not distinguish between these possibilities, and future studies using chaperone-dead or EIF4G-binding-deficient HSP27 mutants will be required to dissect these mechanisms.

Proteotoxic stress is tightly linked to SG formation, which serves as an adaptive mechanism to transiently suppress translation under stress conditions. Our SG proteomic analysis revealed extensive remodeling of SG composition following HSP27 depletion, including increased sequestration of key translational regulators such as EIF4E, EIF3M, EIF4A2, EIF2S2, mTOR, and QKI within SG fractions. These results suggest that HSP27 loss is associated with enhanced compartmentalization of translation-related proteins into SGs, which may reinforce translational arrest. Given that HSP27 has been reported to directly associate with translation initiation factors such as EIF4G, it is plausible that HSP27 influences global translation through both SG-dependent and SG-independent mechanisms; however, distinguishing these pathways will require further investigation.

Beyond SG dynamics, emerging evidence suggests crosstalk between HSP27 and the ubiquitination machinery, as we observed in our ubiquitomics analysis [59]. Whether HSP27 directly regulates the ubiquitination status of key translation factors, or whether the observed ubiquitination changes are secondary consequences of altered translation and protein folding, remains to be determined. Nonetheless, HSP27 depletion was associated with extensive ubiquitination of translation-associated proteins, including EIF3B, EIF3G, EIF4A3, and EIF4G1, consistent with destabilization of the translational machinery. Several oncogenic regulators, including AKT2, PARP1, and DDX5, also exhibited significantly increased ubiquitination following HSP27 depletion. Interestingly, multiple proteasome-associated subunits were enriched within SG fractions following HSP27 depletion, raising the possibility of functional coupling between SG remodeling and ubiquitin-mediated protein turnover during proteotoxic stress, although the nature of this coupling remains speculative at this stage.

From a therapeutic perspective, our findings associate proteostasis dependence with a potential vulnerability in glioblastoma, IDH-wildtype. However, the therapeutic implications of this vulnerability must be interpreted within an appropriate conceptual framework: HSP27 does not operate as a mutationally activated oncogenic driver that single-agent targeting can fully disable (e.g., EGFR mutants in lung adenocarcinoma), but rather as a stress-inducible chaperone that confers a buffering capacity against proteotoxic burden. This distinction is particularly relevant in the hostile glioblastoma microenvironment, where hypoxia, nutrient deprivation, and therapy-induced stress prevail. HSP27 depletion alone lowers the proteotoxic threshold without triggering cell death, which explains the modest efficacy of OGX-427 monotherapy in our orthotopic models. It does not abolish malignant capacity outright, as glioblastoma cells can engage alternative adaptive mechanisms. Consequently, we reasoned that HSP27 inhibition may be most effective when combined with agents that impose additional proteotoxic stress. Indeed, pharmacological inhibition of HSP27 using OGX-427 significantly enhanced the antitumor efficacy of the translational inhibitor Didemnin B in both in vitro and orthotopic glioblastoma models, with the combination reducing tumor progression and prolonging survival compared with either single agent. However, the rationale for this combination warrants careful consideration. HSP27 is not a classical oncogenic driver but rather a stress-adaptive buffer; its depletion alone reduces the proteotoxic threshold without triggering cell death, which may explain the modest single-agent effect we observed. The addition of Didemnin B, which directly impairs protein synthesis, may further elevate proteotoxic burden, potentially pushing glioblastoma cells beyond the collapse point. This conceptual framework supports the combination strategy, yet several uncertainties remain. First, it is unclear whether the enhanced efficacy reflects interaction between the two agents or additive toxicity from independent pathways. Second, both agents may have distinct toxicity profiles in normal tissues—particularly the brain, where translational inhibition could affect neuronal function—and our study did not systematically evaluate these risks. Third, while OGX-427 has shown acceptable safety in clinical trials for other solid tumors, and Didemnin B has undergone prior clinical evaluation, their combination has not been tested in humans; thus, the therapeutic window and potential overlapping toxicities remain unknown. These considerations underscore the need for dedicated pharmacokinetic and toxicological studies before clinical translation.

Nevertheless, several limitations should be acknowledged. First, our study focused on established glioblastoma cell lines and orthotopic models; the extent to which these findings apply to patient-derived tumors with heterogeneous HSP27 expression levels requires further validation. Second, we acknowledge that the U87-MG cell line used in our orthotopic models has well-documented limitations as a representative glioblastoma model, as commercially available U87-MG may differ from the original tumor-derived line and may not fully capture the genetic heterogeneity of patient-derived GBM. To mitigate this, we validated our key in vitro findings—including drug combination effects—in both U87-MG and U251 cells, with consistent results. Third, the unbiased Ribo-seq/RNA-seq analyses were performed using a single HSPB1-targeting siRNA, and these datasets were not repeated with a second independent siRNA or an orthogonal perturbation strategy; however, the core phenotypes were independently validated using sgRNA-mediated knockout in U87 and shRNA-mediated knockdown in U251. Fourth, the potential off-target effects of combining translational inhibition with HSP27 blockade on normal brain cells were not systematically evaluated, which is critical for assessing the therapeutic window. Fifth, claims regarding blood–brain barrier penetration should be interpreted with caution, as efficiency is context-dependent and may vary with administration route, formulation, and tumor-induced blood–brain barrier disruption. Mechanistically, dissecting the temporal order of translational arrest, ubiquitination changes, and stress-granule dynamics upon dual inhibition would help establish causality. Additionally, understanding how tumor cells adapt—or fail to adapt—to the simultaneous collapse of translation and chaperone networks will be crucial for developing resistance-preventing strategies.

## 5. Conclusions

In summary, this study characterizes HSP27 as a key regulator associated with translational control, proteostasis maintenance, stress adaptation, and ubiquitinome remodeling in glioblastoma, IDH-wildtype. Targeting HSP27 reduced tumor progression and prolonged survival in orthotopic models, supporting HSP27-dependent proteostasis buffering as a potential therapeutic vulnerability in this entity.

## Data Availability

The mass spectrometry proteomics data have been deposited to the ProteomeXchange Consortium (https://proteomecentral.proteomexchange.org) via the iProX partner repository [1,2] with the dataset identifier PXD079961.

## Supplementary Materials

The following supporting information can be downloaded at: https://www.mdpi.com/article/10.3390/cells15191794/s1, Figure S1: **HSP27 promotes glioblastoma cell proliferation, migration and tumor growth.** (A) Kaplan–Meier overall survival curves for patients with glioblastoma, IDH-wildtype (GBM) from the Gravendeel dataset, stratified by HSPB1 expression level (upper quartile cutoff). High-expression group: n = 45, median survival = 10.9 months; low-expression group: n = 136, median survival = 15.5 months; HR = 0.69, 95% CI 0.49–0.98 (low vs. high expression); log-rank *p* = 0.0356. (B–D) Immunoblotting analysis confirming *HSPB1* knockdown in U87-MG and U251-MG glioblastoma cells. (E) CCK-8 assays assessing the proliferative capacity of U251-MG cells following *HSPB1* knockdown. Statistical significance was determined using two-way ANOVA followed by Šidák’s post hoc test. (F) Transwell migration assays evaluating the migratory ability of U251-MG cells following *HSPB1* knockdown. Statistical significance was determined using a two-tailed unpaired Student’s *t*-test. (G) Immunoblot analysis confirming stable *HSPB1* knockdown in U87-MG glioblastoma cells. (H) CCK-8 assays assessing the proliferative capacity of U87-MG cells following *HSPB1* knockdown. Statistical significance was determined by two-way ANOVA. (I) Transwell migration assays evaluating the migratory ability of U87-MG cells following *HSPB1* knockdown. Statistical significance was determined by Unpaired *t* test. (J) Immunoblot analysis confirming stable HSP27 overexpression in U87-MG glioblastoma cells. (K) CCK-8 assays assessing the proliferative capacity of U87-MG cells following HSP27 overexpression. Statistical significance was determined using two-way ANOVA followed by Šidák’s post hoc test. (L) Transwell migration assays evaluating the migratory ability of U87-MG cells following HSP27 overexpression. Statistical significance was determined by Unpaired *t* test. Data information: Data are presented as mean ± SEM unless otherwise stated. *n* represents the number of independent biological replicates or mice as indicated. *p* < 0.05 was considered statistically significant. ns means not significant; * means *p* < 0.05. ** means *p* < 0.01.*** means *p* < 0.001. HR, hazard ratio; CI, confidence interval.; Figure S2: **Immunohistochemical validation of *HSPB1* knockdown in mouse brain tumors.** (A) Immunoblot analysis validating CRISPR-Cas9-mediated *HSPB1* knockout in luciferase-expressing U87-MG cells. (B) Quantification of bioluminescence signals from luciferase-expressing U87-MG cells using a microplate reader, confirming stable luciferase expression. Data are presented as mean ± SEM from three independent biological replicates. Statistical significance was determined by Unpaired *t* test, * means *p* < 0.05. ** means *p* < 0.01.*** means *p* < 0.001. Representative IHC images of HSP27 in brain sections from mice bearing WT U87-MG-Luc tumors or HSP27-KO U87-MG-Luc tumors. Tumor tissues were harvested at the humane endpoint (28 days post-implantation). Figure S3: **HSP27 regulates protein translation and cell-cycle progression in glioblastoma cells.** (A) Immunoblot analysis of puromycin incorporation showing global protein synthesis efficiency in U251 cells after HSP27 depletion. (B) Immunoblot analysis showing reduced protein expression of SKP2, CDK2, NQO1, GPX8, FABP5, and SNCG proteins in U251 cells after HSP27 depletion. Experiments in C were repeated at least three times. (C) Quantification of the relative protein expression level. (D) qPCR analysis of SKP2, CDK2, NQO1, GPX8, FABP5, and SNCG mRNA levels. (E) Scatter plot showing protein abundance ratios from the CPTAC GBM dataset (n ≈ 99). A significant positive correlation was observed (Spearman r = 0.44, *p* = 5.072 × 10^−6^). (F) Flow cytometric analysis of cell-cycle distribution in U251 cells after *HSPB1* knockdown. The right graph is the quantification of the three flow cytometry replicates shown on the left. Data are presented as mean ± SEM from three independent biological replicates. Statistical significance was determined by Unpaired *t* test, * means *p* < 0.05. ** means *p* < 0.01.*** means *p* < 0.001. Figure S4: **HSP27 facilitates protein folding and prevents unfolded-protein accumulation in glioblastoma cells.** (A) Western blot analysis confirming efficient knockdown of *HSPB1* in glioblastoma cells. (B,C) Western blot analysis of HSPs in U87-MG and U251-MG cells upon HSP27 depletion. (D) Immunoblot analysis of the indicated proteins in soluble fractions separated by SDS-PAGE. (E–H) Immunoblot analysis of LAMTOR2, CLN3, CALCOCO1 and FAK in the soluble fractions of cell lysates separated by native PAGE. Table S1: shRNA, siRNA and OGX-427 sequences used in this study; Table S2: Primers used in qPCR; Table S3: Antibodies used in this study.
