# Supplementary material for: HSP27 Provides Stress-Adaptive Proteostasis Buffering in Glioblastoma and Is Therapeutically Vulnerable
Source: Cells. 2026 Sep 30;15(19):1794. doi: 10.3390/cells15191794 (PMC13664483; doi:10.3390/cells15191794)
Supplement: Supplementary file 1 [file cells-15-01794-s001.zip › cells-4549703-supplementary.pdf]

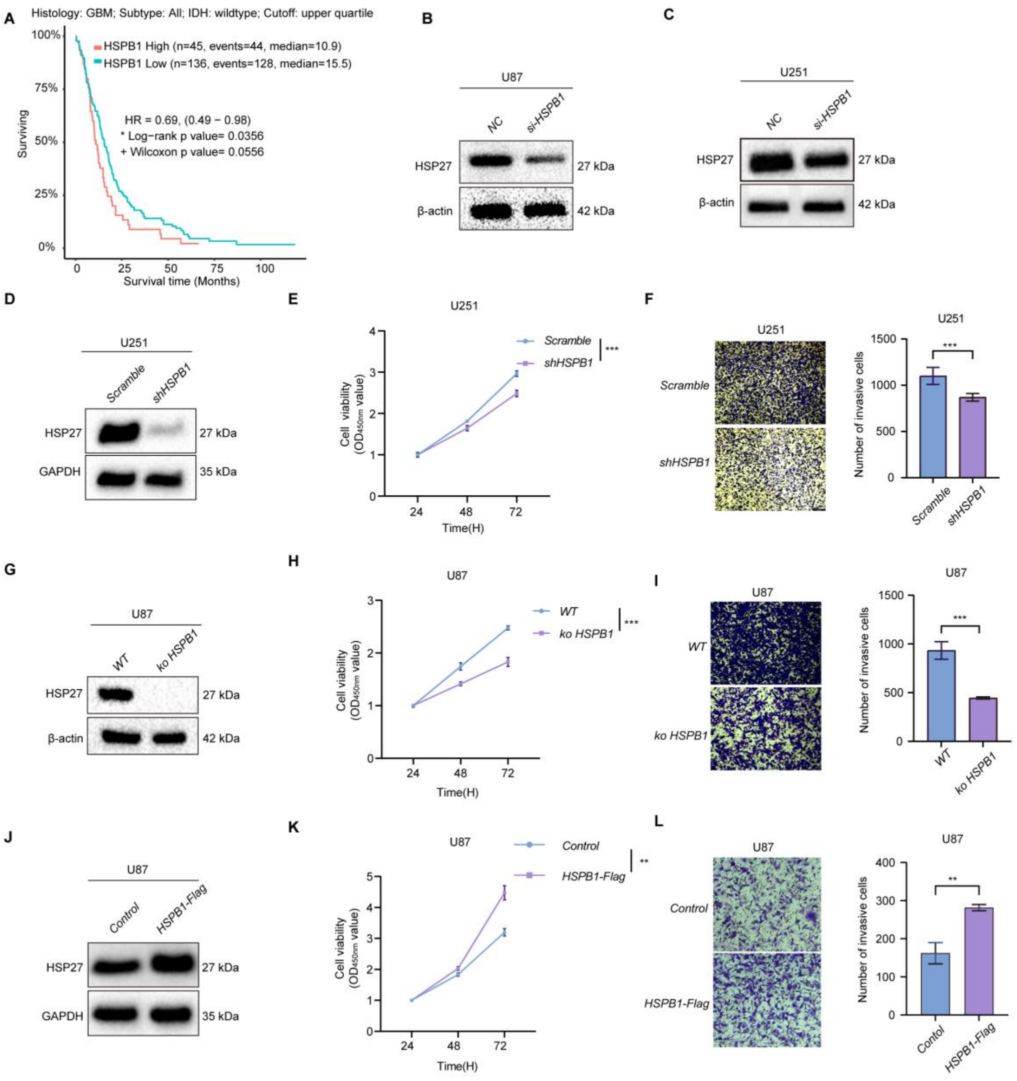

Supplementary Figure S1 HSP27 promotes glioblastoma cell proliferation, migration and tumor growth. A) Kaplan–Meier overall survival curves for patients with glioblastoma, IDH-wildtype (GBM) from the Gravendeel dataset, stratified by HSPB1 expression level (upper quartile cutoff). High-expression group: n = 45, median survival = 10.9 months; low-expression group: n = 136, median survival = 15.5 months; HR = 0.69, 95% CI 0.49–0.98 (low vs. high expression); log-rank P = 0.0356. B-D) Immunoblotting analysis confirming *HSPB1* knockdown in U87-MG and U251-MG glioblastoma cells. E) CCK-8 assays assessing the proliferative capacity of U251-MG cells following *HSPB1* knockdown. Statistical significance was determined using two-way ANOVA followed by Šidák's post hoc test. F) Transwell migration assays evaluating the migratory ability of U251-MG cells following *HSPB1* knockdown. Statistical significance was determined using a two-tailed unpaired Student's t-test. G) Immunoblot analysis confirming stable *HSPB1* knockdown in U87-MG glioblastoma cells. H) CCK-8 assays assessing the proliferative capacity of U87-MG cells following *HSPB1* knockdown. Statistical significance was determined by two-way ANOVA. I) Transwell migration assays evaluating the migratory ability of U87-MG cells following *HSPB1* knockdown. Statistical significance was determined by Unpaired t test. J) Immunoblot analysis confirming stable HSP27 overexpression in U87-MG glioblastoma cells. K) CCK-8 assays assessing the proliferative capacity of U87-MG cells following HSP27 overexpression. Statistical significance was

determined using two-way ANOVA followed by Šidák's post hoc test. L) Transwell migration assays evaluating the migratory ability of U87-MG cells following HSP27 overexpression. Statistical significance was determined by Unpaired t test. Data information: Data are presented as mean ± SEM unless otherwise stated. *n* represents the number of independent biological replicates or mice as indicated. $P < 0.05$ was considered statistically significant. ns means not significant; * means $P < 0.05$. ** means $P < 0.01$.*** means $P < 0.001$. HR, hazard ratio; CI, confidence interval.

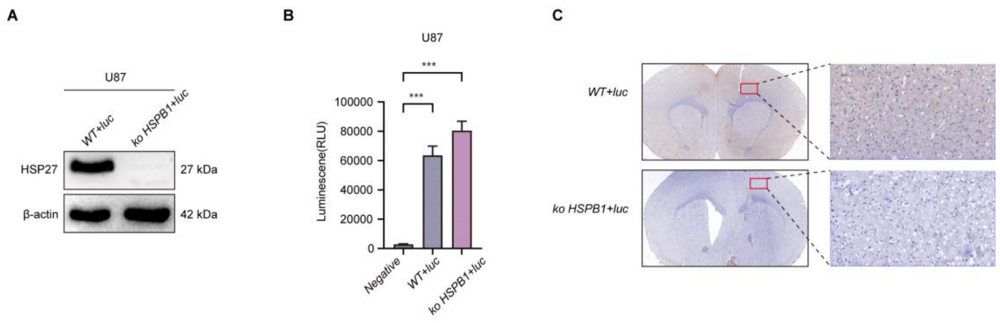

Supplementary Figure S2 Immunohistochemical validation of *HSPB1* knockdown in mouse brain tumors. A) Immunoblot analysis validating CRISPR-Cas9-mediated *HSPB1* knockout in luciferase-expressing U87-MG cells. B) Quantification of bioluminescence signals from luciferase-expressing U87-MG cells using a microplate reader, confirming stable luciferase expression. Data are presented as mean ± SEM from three independent biological replicates. Statistical significance was determined by Unpaired t test, * means $P < 0.05$. ** means $P < 0.01$.*** means $P < 0.001$. Representative IHC images of HSP27 in brain sections from mice bearing WT U87-MG-Luc tumors or HSP27-KO U87-MG-Luc tumors. Tumor tissues were harvested at the humane endpoint (28 days post-implantation).

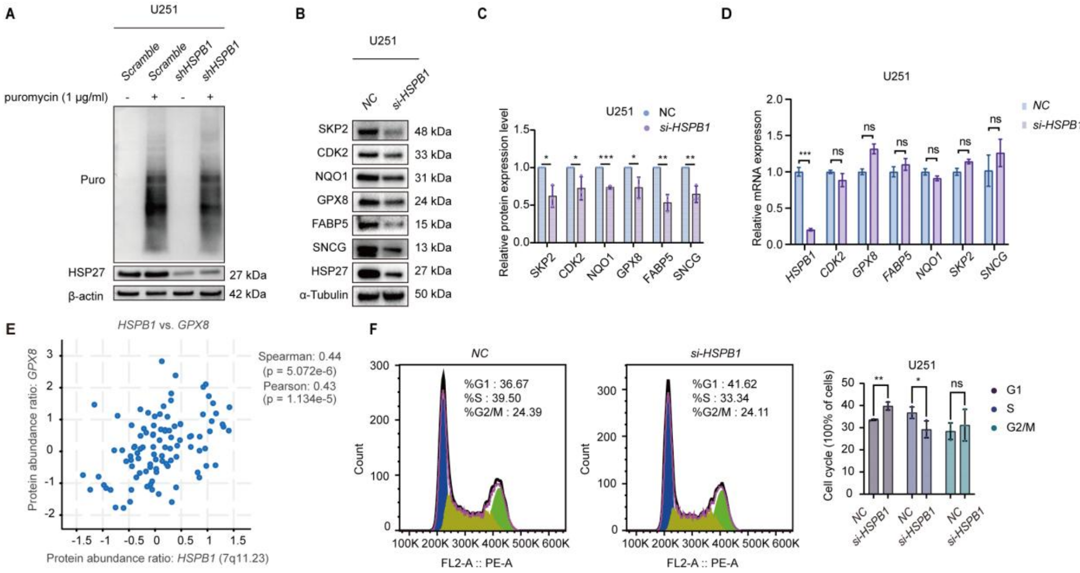

Supplementary Figure S3 HSP27 regulates protein translation and cell-cycle progression in glioblastoma cells. A) Immunoblot analysis of puromycin incorporation showing global protein synthesis efficiency in U251 cells after HSP27 depletion. B) Immunoblot analysis showing reduced protein expression of SKP2, CDK2, NQO1, GPX8, FABP5, and SNCG proteins in U251 cells after HSP27 depletion. Experiments in C

were repeated at least three times. C) Quantification of the relative protein expression level. D) qPCR analysis of SKP2, CDK2, NQO1, GPX8, FABP5, and SNCG mRNA levels. E) Scatter plot showing protein abundance ratios from the CPTAC GBM dataset (n≈99). A significant positive correlation was observed (Spearman r = 0.44, P = 5.072e-6). F) Flow cytometric analysis of cell-cycle distribution in U251 cells after *HSPB1* knockdown. The right graph is the quantification of the three flow cytometry replicates shown on the left. Data are presented as mean ± SEM from three independent biological replicates. Statistical significance was determined by Unpaired t test, * means $P < 0.05$. ** means $P < 0.01$.*** means $P < 0.001$.

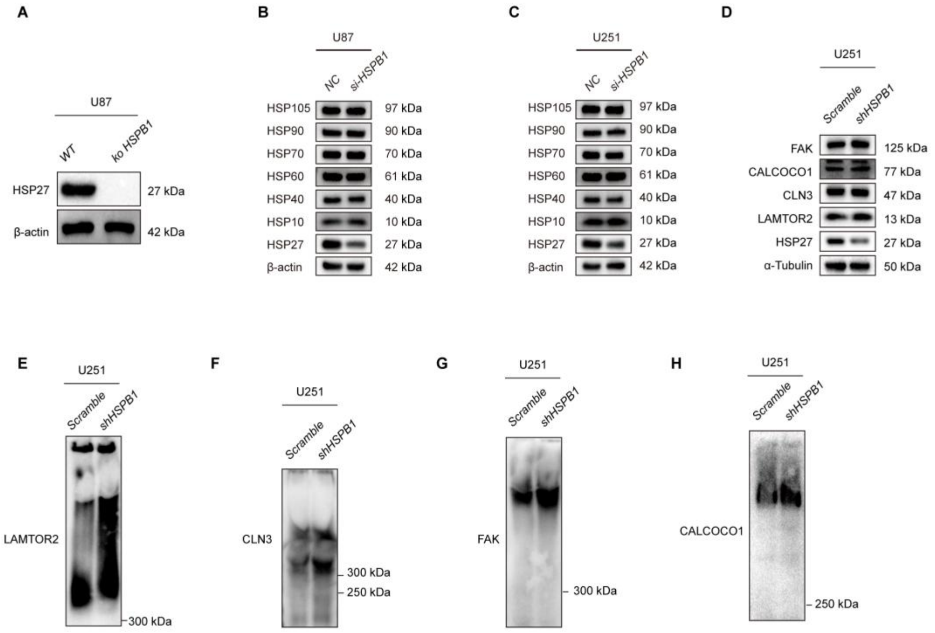

Supplementary Figure S4 HSP27 facilitates protein folding and prevents unfolded-protein accumulation in glioblastoma cells. A) Western blot analysis confirming efficient knockdown of *HSPB1* in glioblastoma cells. B, C) Western blot analysis of HSPs in U87-MG and U251-MG cells upon HSP27 depletion. D) Immunoblot analysis of the indicated proteins in soluble fractions separated by SDS-PAGE. E-H) Immunoblot analysis of LAMTOR2, CLN3, CALCOCO1 and FAK in the soluble fractions of cell lysates separated by native PAGE.

Supplementary Table S1. shRNA, siRNA and OGX-427 sequences used in this study

Name	Sequence (5′→3′)
si-HSP27	GUUCUUUUGAUACAUUUAUCU
OGX-427	GGGACGCGGCGCTCGGTCAT
shHSP27	GATCACCATCCCAGTCACCTT
sgHSP27	GGATGTCAACCACTTCGC
NC	UUCUCCGAACGUGUCACGU

Supplementary Table S2. Primers used in qPCR

Name	Forward Sequence (5′→3′)	Reverse Sequence (5′→3′)
Human *HSP27*	CTGACGGTCAAGACCAAGGATG	GTGTATTTCCGCGTGAAGCACC
Human *CDK2*	ATGGATGCCTCTGCTCTCACTG	CCCGATGAGAATGGCAGAAAGC
Human *FABP5*	GGTGCATTGGTTCAGCATCAGG	TCATAGATCCGAGTACAGGTGAC
Human *SKP2*	GATGTGACTGGTCGGTTGCTGT	GAGTTCGATAGGTCCATGTGCTG
Human *GPX8*	CTAGGATCTGAAGGAGAACCTGC	GCCTGATGACTTCAATGGGCTC
Human *SNCG*	TGTGGTGAGCAGCGTCAACACT	TTGGATGCCACACCCTCCTGTT
Human *NQO1*	CCTGCCATTCTGAAAGGCTGGT	GTGGTGATGGAAAGCACTGCCT
Human *GAPDH*	GTCTCCTCTGACTTCAACAGCG	ACCACCCTGTTGCTGTAGCCAA

Supplementary Table S3. Antibodies used in this study

Antibodies	Source	Identifier	WB	IP	IHC
HSP27	Cell Signaling Technology	95357	1:1000		1:50
CDK2	Selleck	F4119	1:1000		
SKP2	Absea	KC-3343	1:1000		
FABP5	Selleck	F1429	1:500		
Ubiqutination	Cell Signaling Technology	20326	1:1000		
Puromycin	Abclonal	A23031	1:1000		
MTOR	Cell Signaling Technology	2983	1:1000		
NQO1	Abmart	T56710F	1:1000		
G3BP1	Selleck	F1559	1:1000		
EIF4E	Selleck	F3587	1:1000		
GPX8	Proteintech	16846-1-AP	1:500		
SNCG	Solarbio	K108130P	1:500		
AKT2	Selleck	F0218	1:1000	1:200	
EIF4G1	Selleck	F0919	1:1000	1:200	
PARP1	Selleck	F0148	1:1000	1:200	
EIF4A3	Selleck	F3433	1:1000	1:200	
DDX5	Proteintech	67025-1-lg	1:1000	1:200	
LAMTOR2	Selleck	F0842	1:1000		
CLN3	Proteintech	67957-1-lg	1:1000		
CALCOCO1	Solarbio	K107717P	1:1000		
FAK	Selleck	F0431	1:1000		
QKI	Selleck	F2558	1:1000		
α-Tubulin	Selleck	F0063	1:10000		
GAPDH	Proteintech	80570-1-RR	1:5000		
β-actin	Abclonal	AC026	1:5000		
